# Supplementary material for: Photocatalytic CO2 reduction to syngas using metallosalen covalent organic frameworks
Source: Nat Commun. 2023 Nov 1;14:6971. doi: 10.1038/s41467-023-42757-7 (PMC10620383; doi:10.1038/s41467-023-42757-7)
Supplement: Supplementary file 1 — Supplementary Information [file 41467_2023_42757_MOESM1_ESM.pdf]

Supporting information for

Photocatalytic CO_2 reduction to syngas using metallosalen covalent organic frameworks

Wei Zhou[1], Xiao Wang[1], Wenling Zhao[1], Naija Lu[1], Die Cong[1], Zhen Li[1], Peigeng Han[1], Guoqing Ren[1], Lei Sun[1], Chengcheng Liu[1,], Wei-Qiao Deng[1,*]*

[1] Institute of Frontier and Interdisciplinary Science, Shandong University, Qingdao, Shandong, 266237, China.

Corresponding Author

Chengcheng Liu: chengcheng.liu@sdu.edu.cn and Wei-Qiao Deng: dengwq@sdu.edu.cn

Chemicals and methods.

Chemicals. All chemicals were purchased from commercial company without further purification. 1, 3, 5-Tri(4-aminophenyl)benzene (purity: 99%) was purchased from Jilin Chinese Academy of Sciences-Yanshen Technology Co., Ltd. 2,4,6-Tris(4-aminophenyl)-1,3,5-triazine (purity: 99%) was purchased from Shanghai Kylpharm Co., Ltd. 4-Hydroxyisophthalaldehyde and 2-hydroxybenzene-1, 3, 5-tricarbaldehyde were purchased from BeiJing Royaltech Co., Ltd. Ethylenediamine, ethanol, cobalt(II) acetate tetrahydrate and zinc acetate dehydrate were purchased from Sinopharm Chemical Reagent Co., Ltd. 1, 2-Dichlorbenzol, triethanolamine, acetonitrile were bought from Shanghai Titan Scientific Co., Ltd. Tris(2, 2′-bipyridyl)dichlororuthenium(II) hexahydrate was bought from Shanghai Aladdin Chemical Reagents.

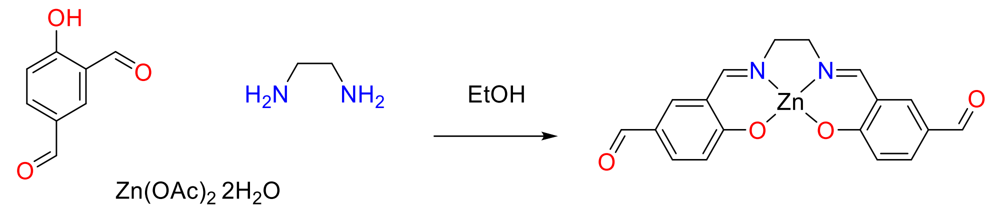

Figure S1. The synthetic process of Zn-CHO.

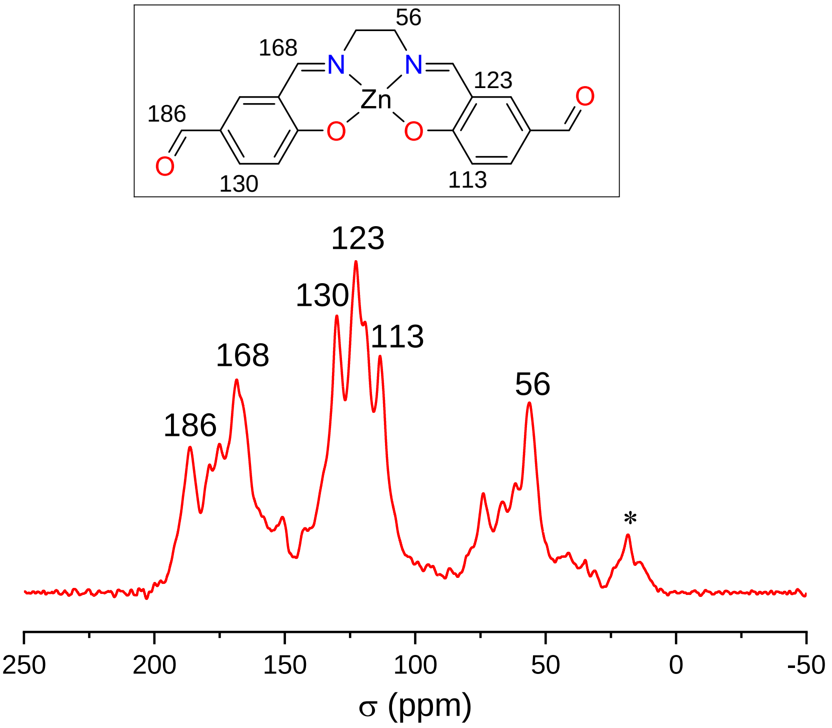

Figure S2. ^{13}C NMR spectrum for Zn-CHO.

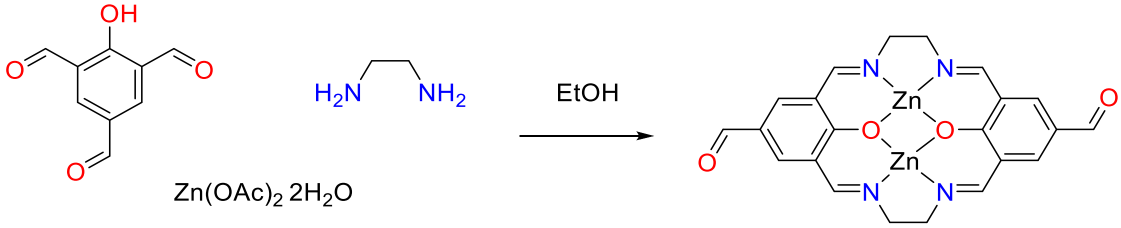

Figure S3. The synthetic process of ZnZn-CHO.

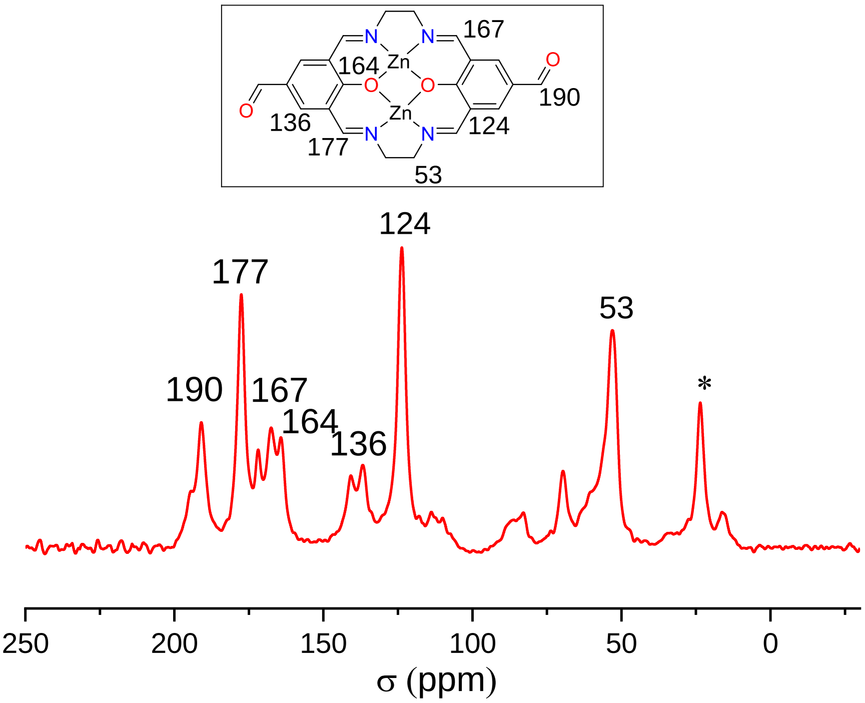

Figure S4. ^{13}C NMR spectrum for ZnZn-CHO.

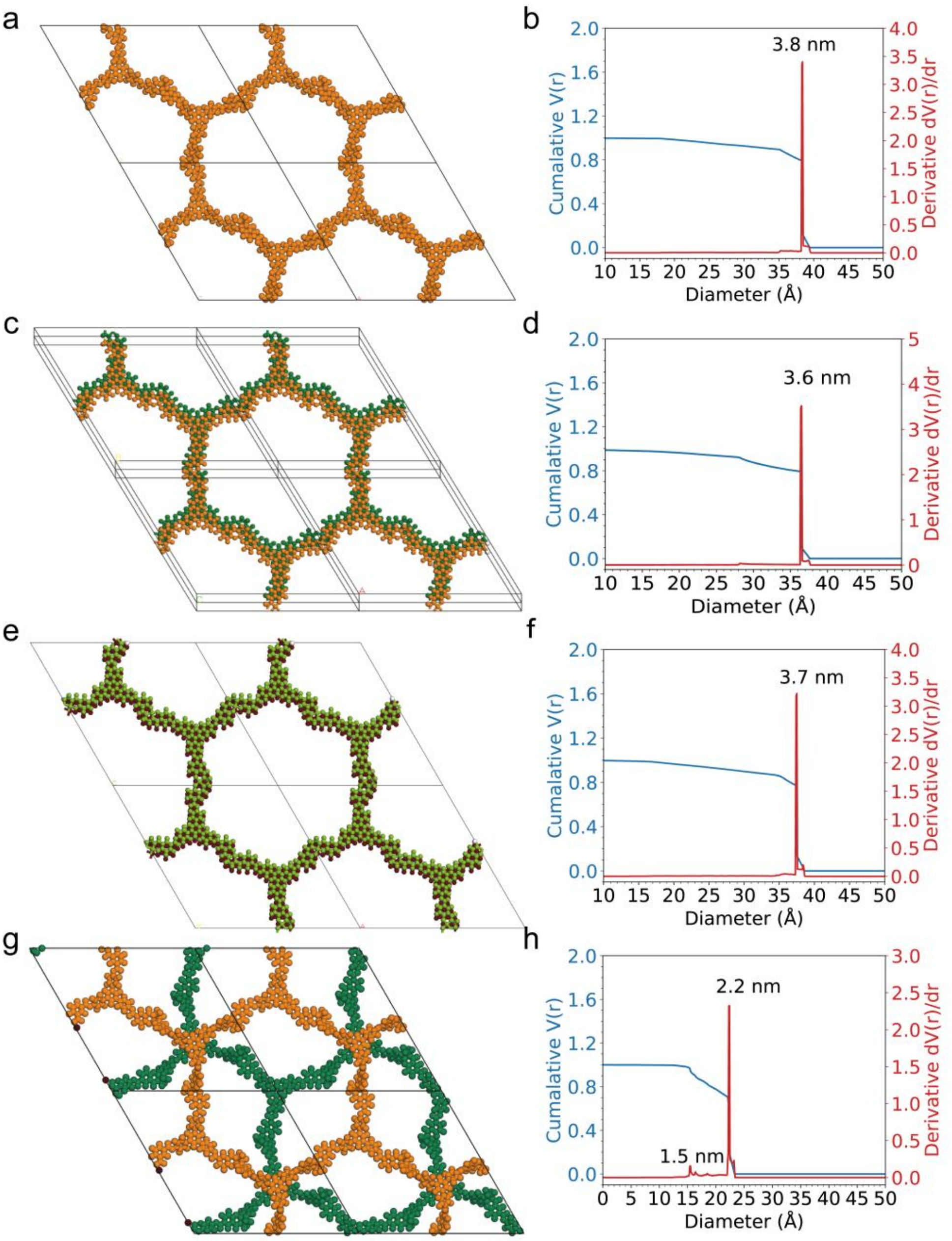

Figure S5. (a, b) Eclipsed AA-stacking model and simulated pore distribution, (c, d) inclined AA-stacking model and simulated pore distribution, (e, f) serrated AA-stacking model and simulated pore distribution, and (g, h) AB-stacking model and simulated pore distribution for Zn-TAPB-COF-1.

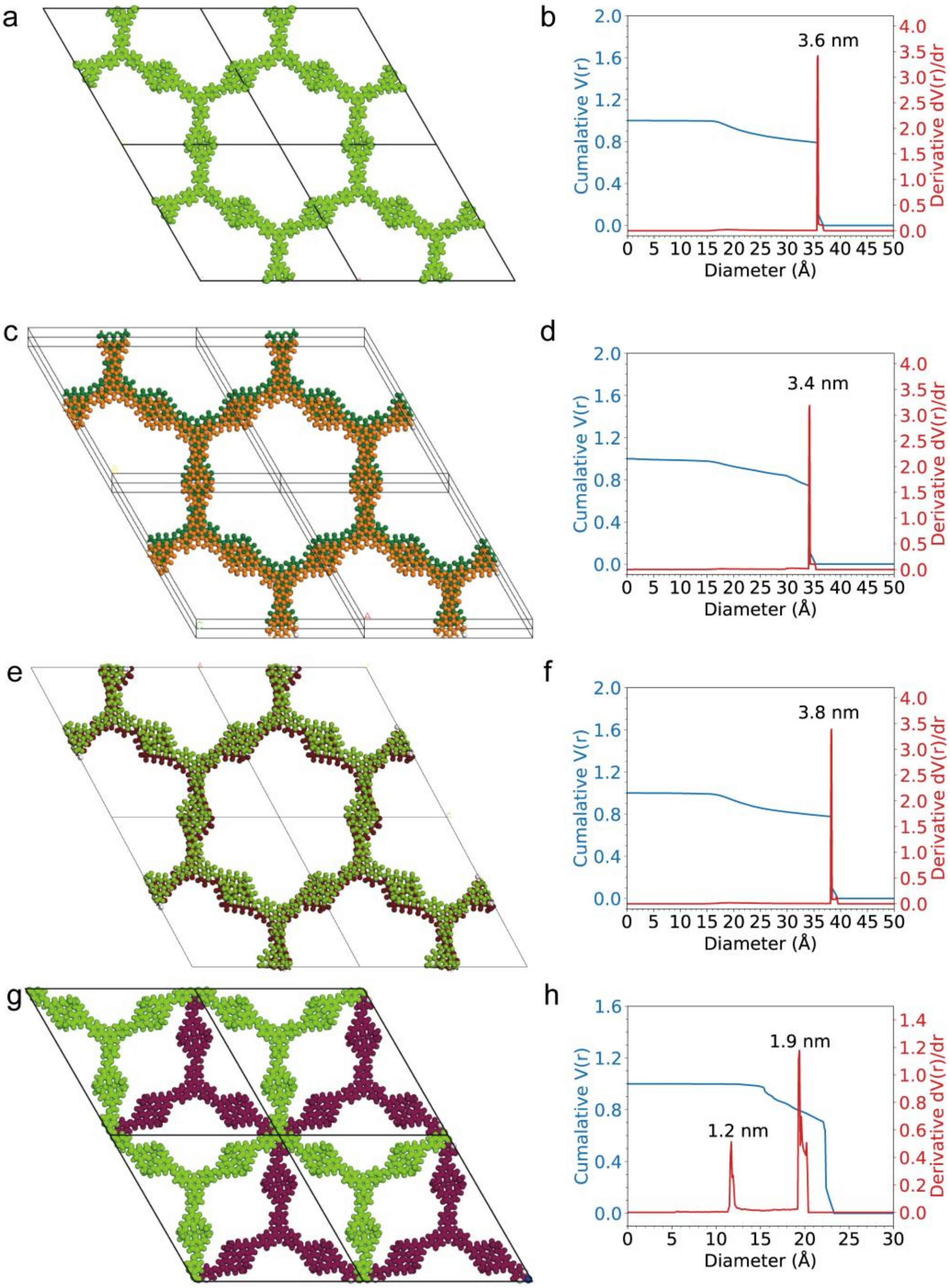

Figure S6. (a, b) Eclipsed AA-stacking model and simulated pore distribution, (c, d) inclined AA-stacking model and simulated pore distribution, (e, f) serrated AA-stacking model and simulated pore distribution, and (g, h) AB-stacking model and simulated pore distribution for ZnZn-TAPB-COF-1.

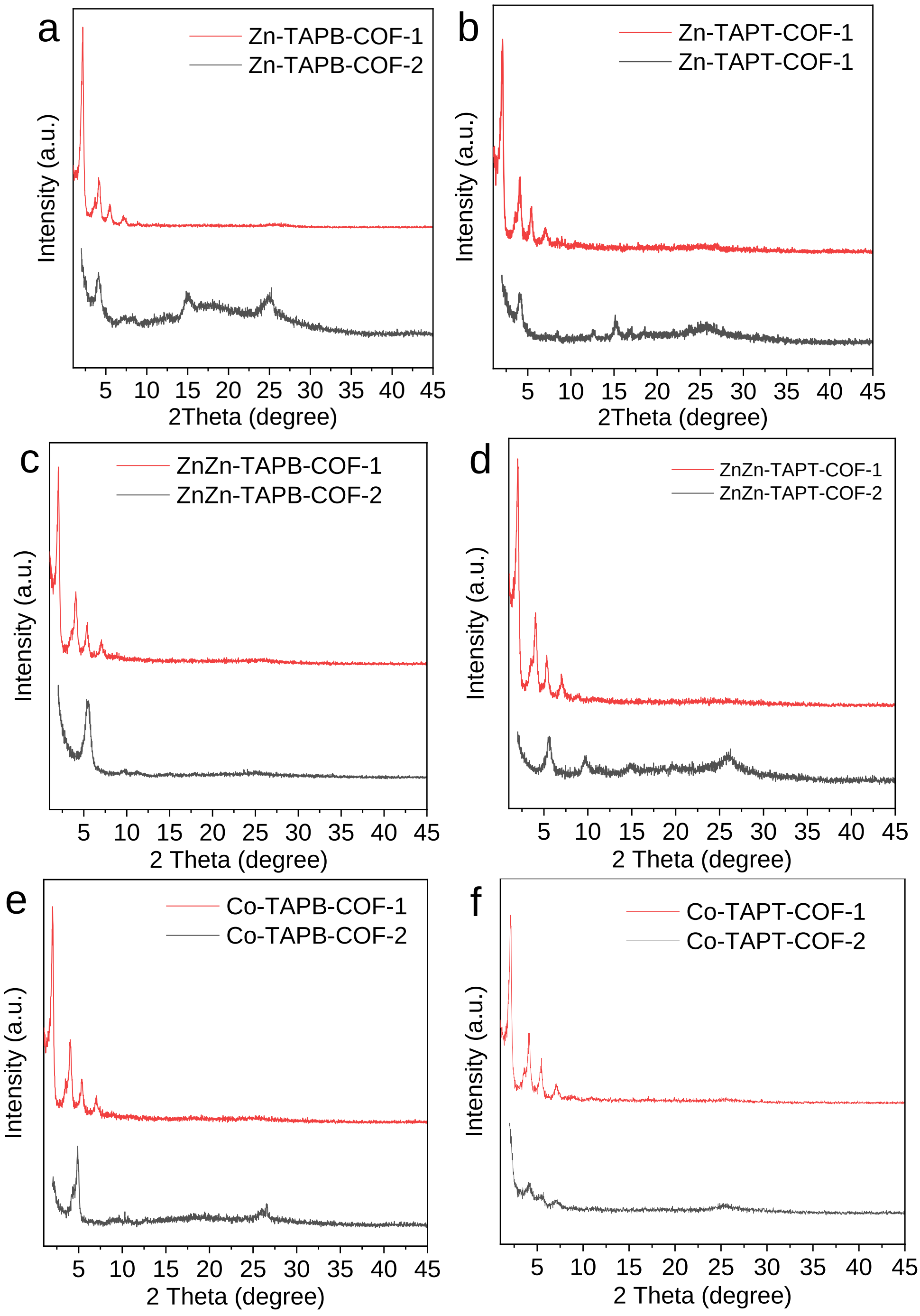

Figure S7. PXRD patterns for (a) Zn-TAPB-COF-(1, 2), (b) Zn-TAPT-COF-(1, 2), (c) ZnZn-TAPB-COF-(1, 2), (d) ZnZn-TAPT-COF-(1, 2), (e) Co-TAPB-COF-(1, 2), (f) Co-TAPT-COF-(1, 2).

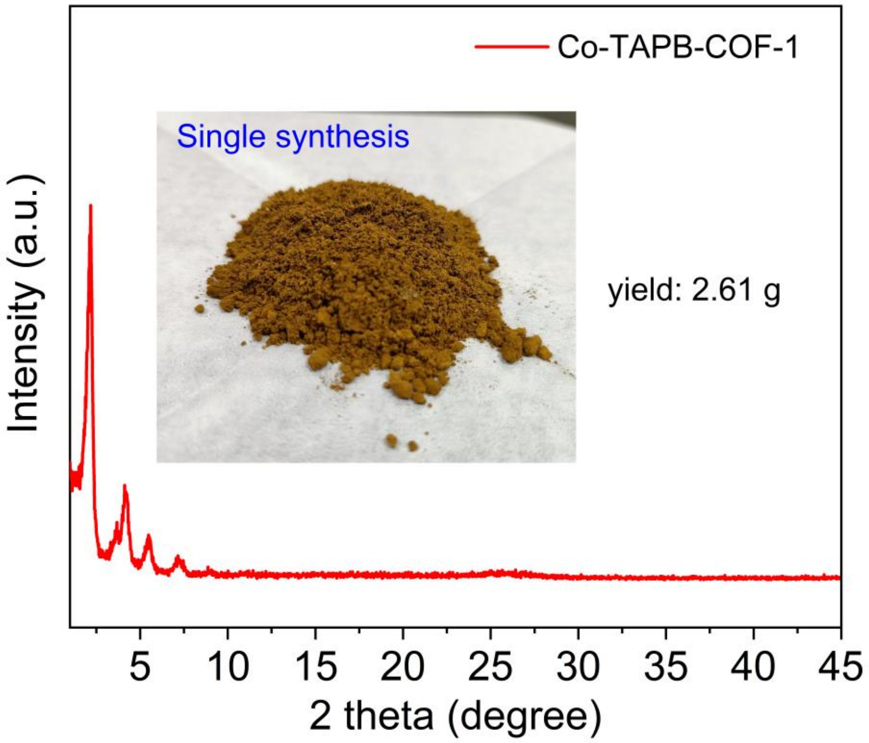

Figure S8. Co-TAPB-COF-1 obtained in autoclave by one-step synthesis without vacuum evacuation.

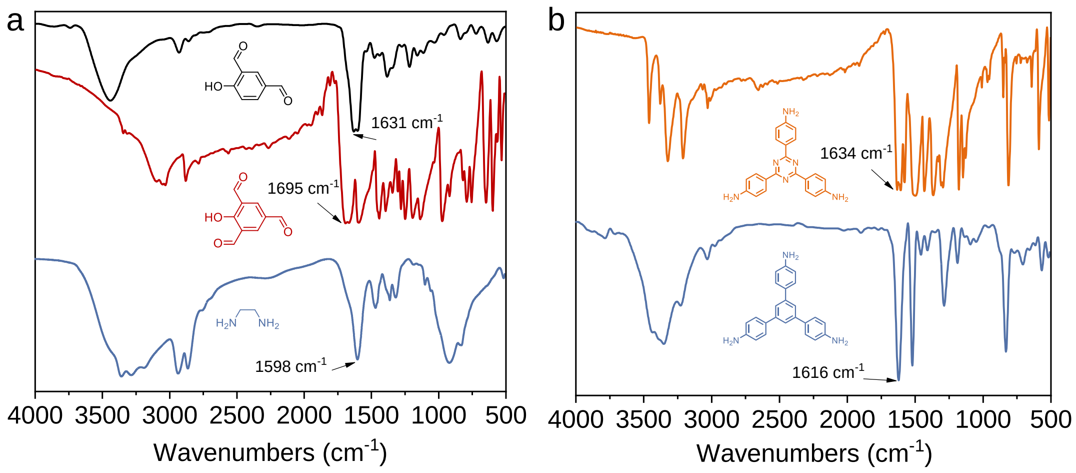

Figure S9. FT-IR spectra of the ligands.

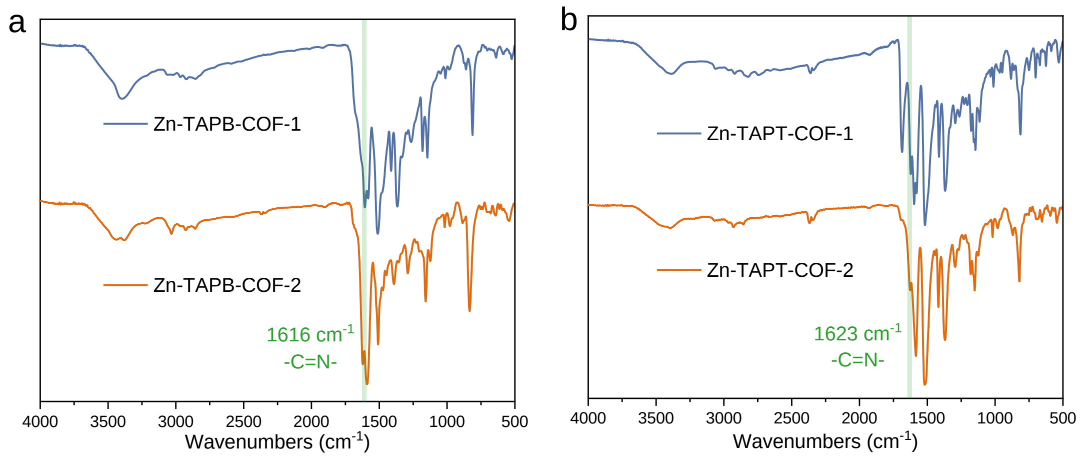

Figure S10. FT-IR spectra of (a) Zn-TAPB-COF-(1, 2) and (b) Zn-TAPT-COF-(1, 2).

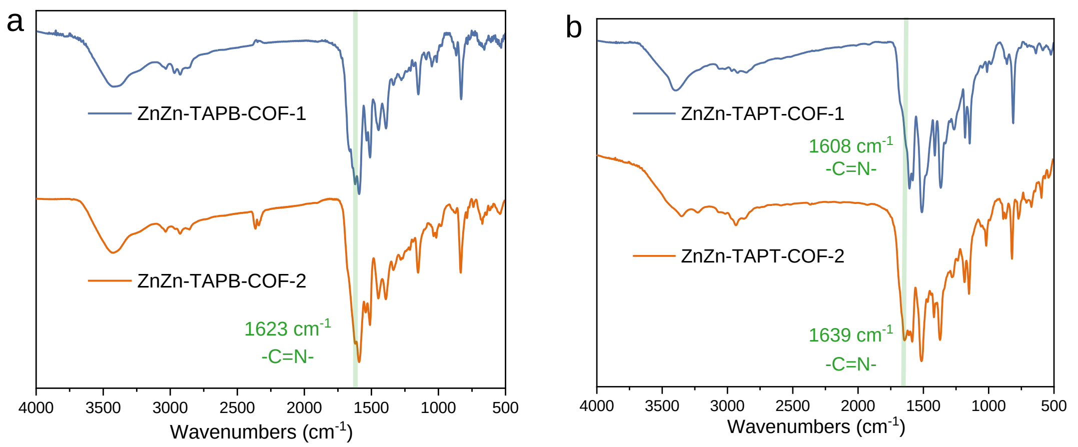

Figure S11. FT-IR spectra of ZnZn-TAPB-COF-(1, 2) and ZnZn-TAPT-COF-(1, 2).

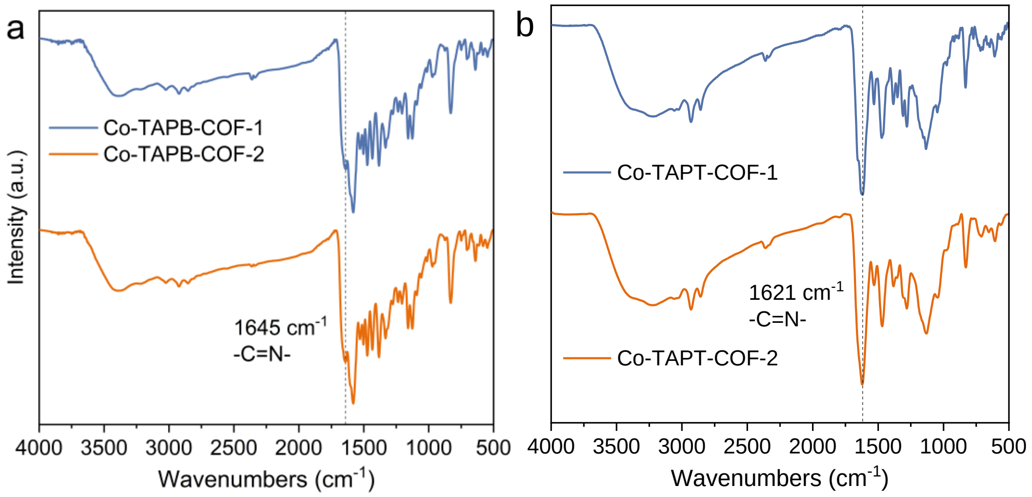

Figure S12. FT-IR spectra of Co-TAPB-COF-(1, 2) and Co-TAPT-COF-(1, 2).

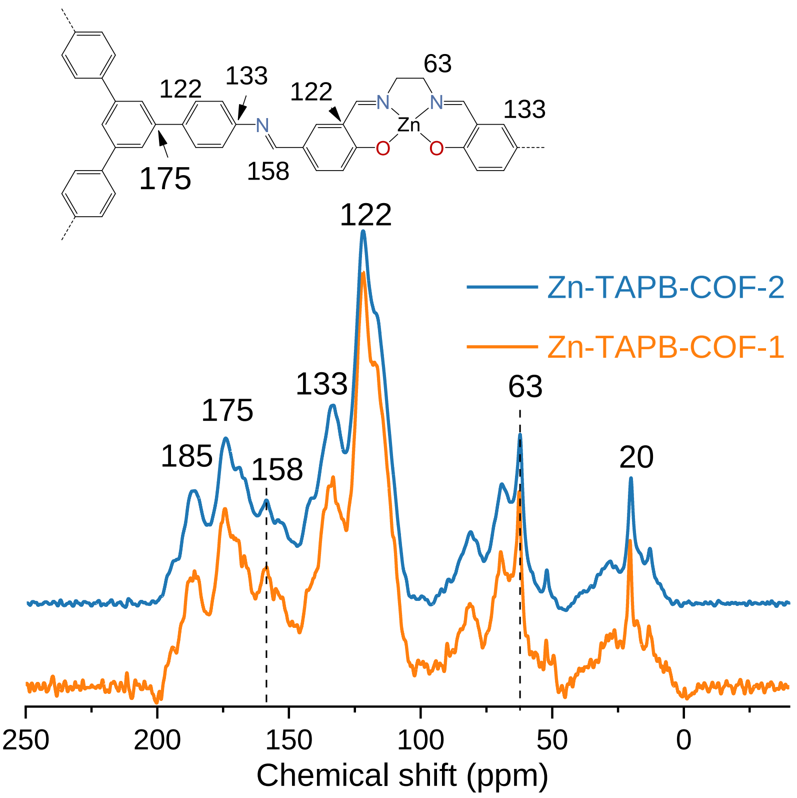

Figure S13. ^{13}C-NMR spectra of Zn-TAPB-COF-(1, 2).

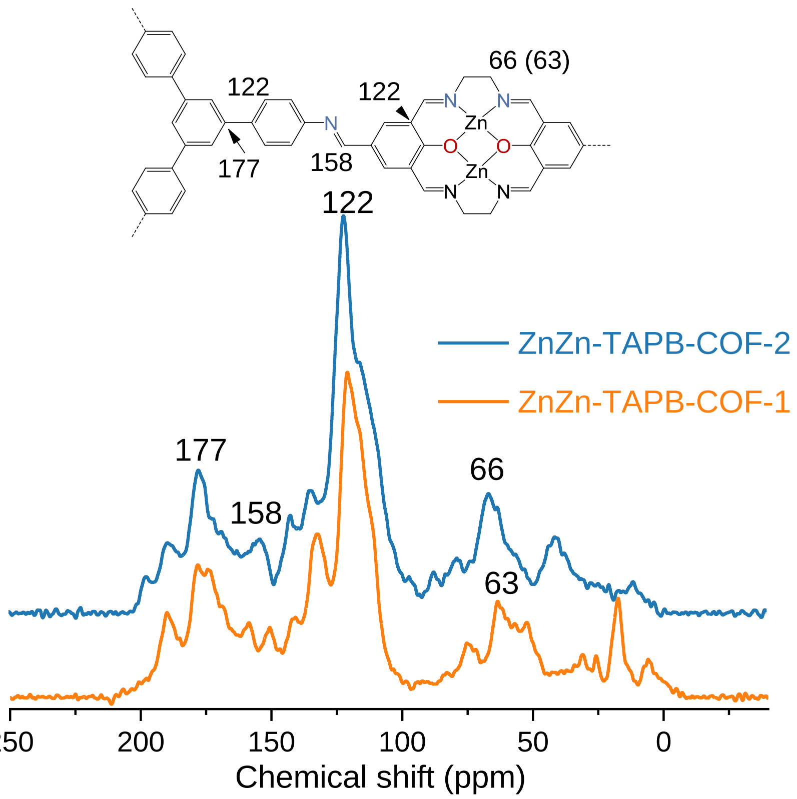

Figure S14. ^{13}C-NMR spectra of ZnZn-TAPB-COF-(1, 2).

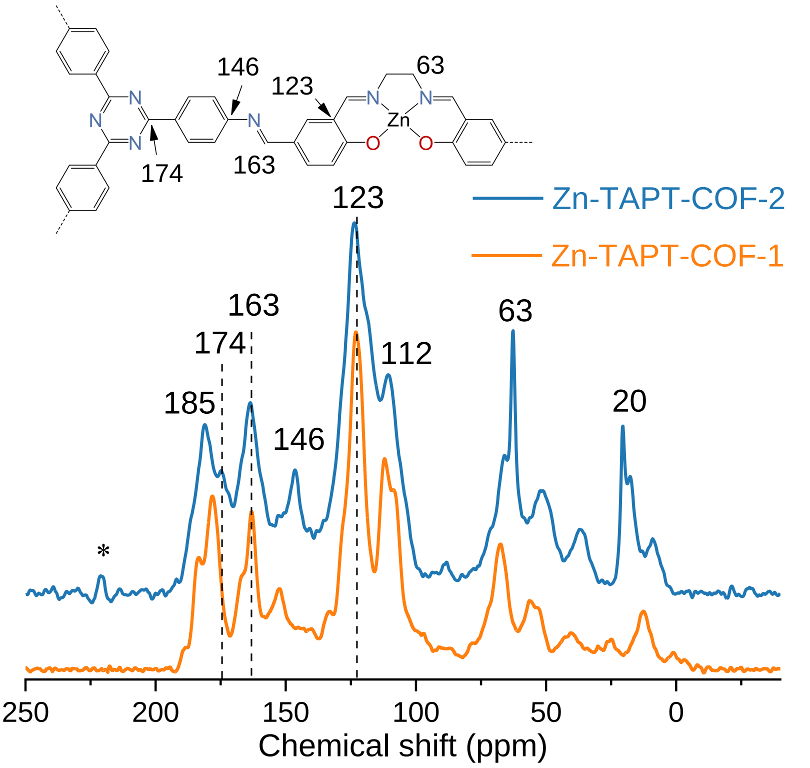

Figure S15. ^{13}C-NMR spectra of Zn-TAPT-COF-(1, 2).

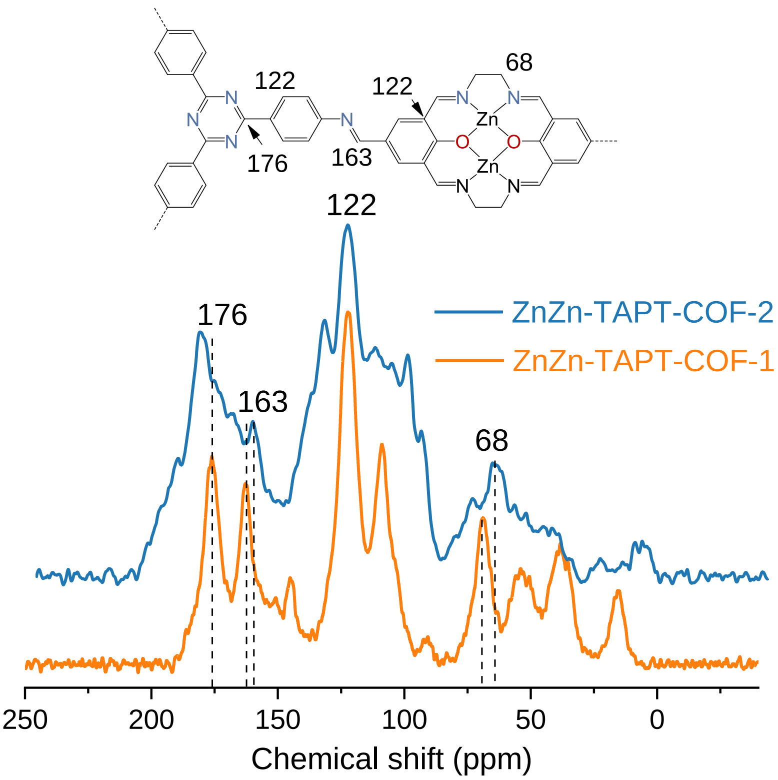

Figure S16. ^{13}C-NMR spectra of ZnZn-TAPT-COF-(1, 2).

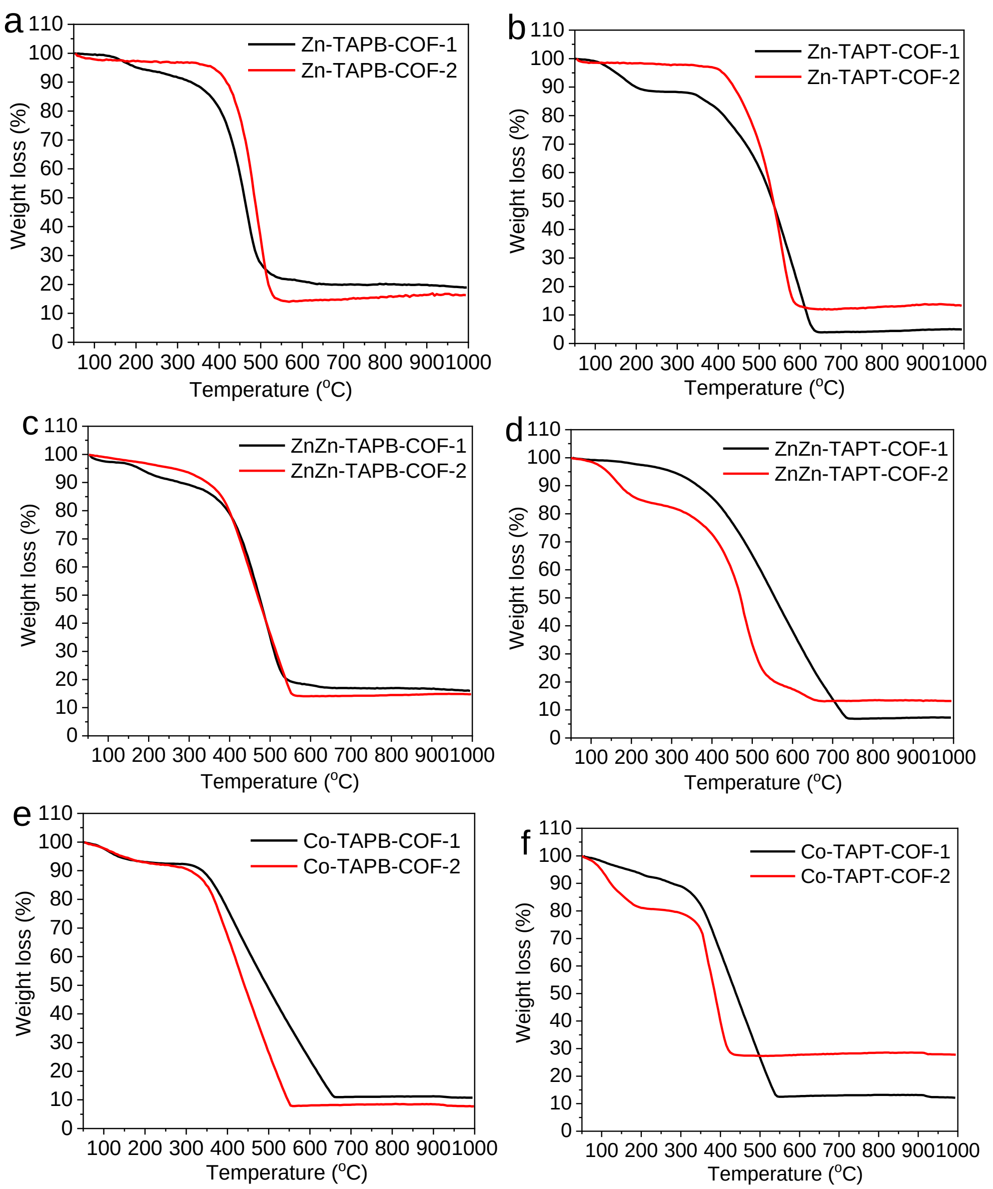

Figure S17. TGA curves of (a) Zn-TAPB-COF-(1, 2), (b) Zn-TAPT-COF-(1, 2), (c) ZnZn-TAPB-COF-(1, 2), (d) ZnZn-TAPT-COF-(1, 2), (e) Co-TAPB-COF-(1, 2) and (f) Co-TAPT-COF-(1, 2).

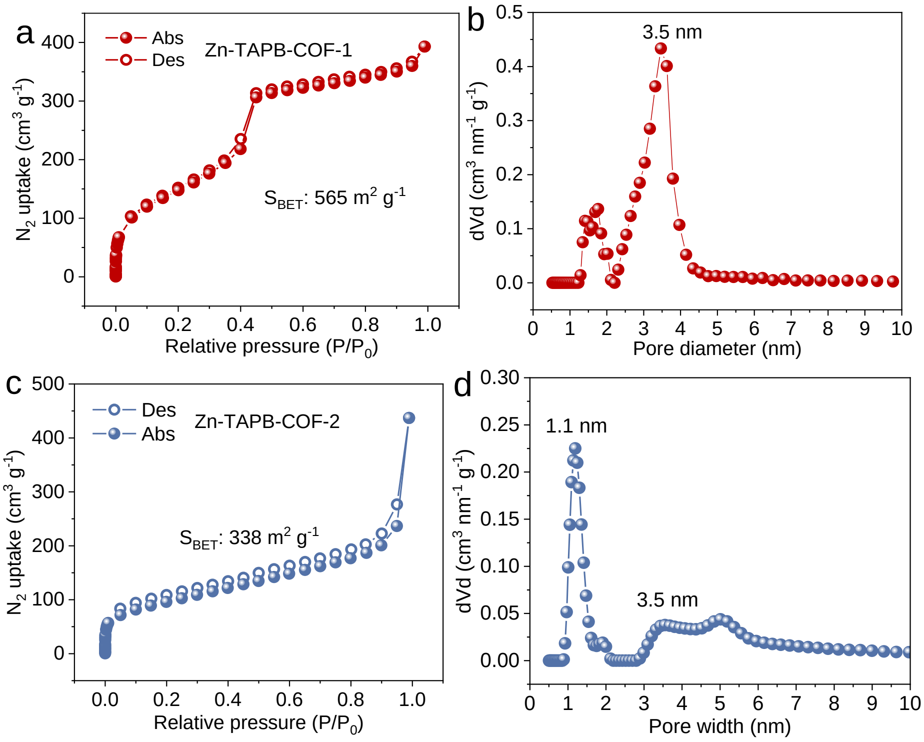

Figure S18. N_2 absorption and desorption isotherms (77 K), pore size distribution for (a, b) Zn-TAPB-COF-1 and (c, d) Zn-TAPB-COF-2.

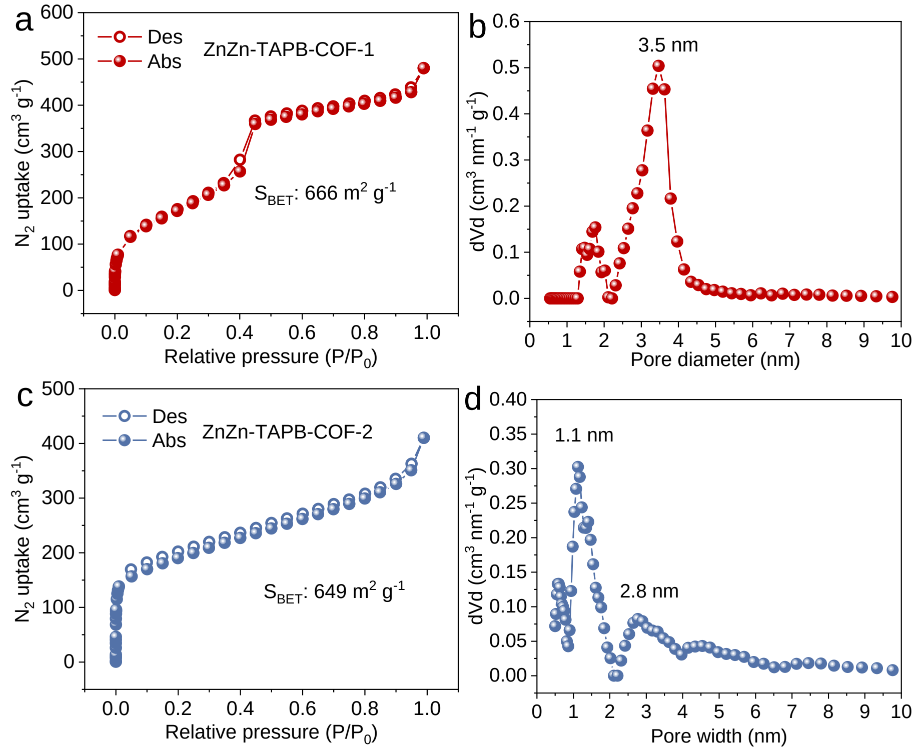

Figure S19. N_2 absorption and desorption isotherms (77 K), pore size distribution for (a, b) ZnZn-TAPB-COF-1 and (c, d) ZnZn-TAPB-COF-2.

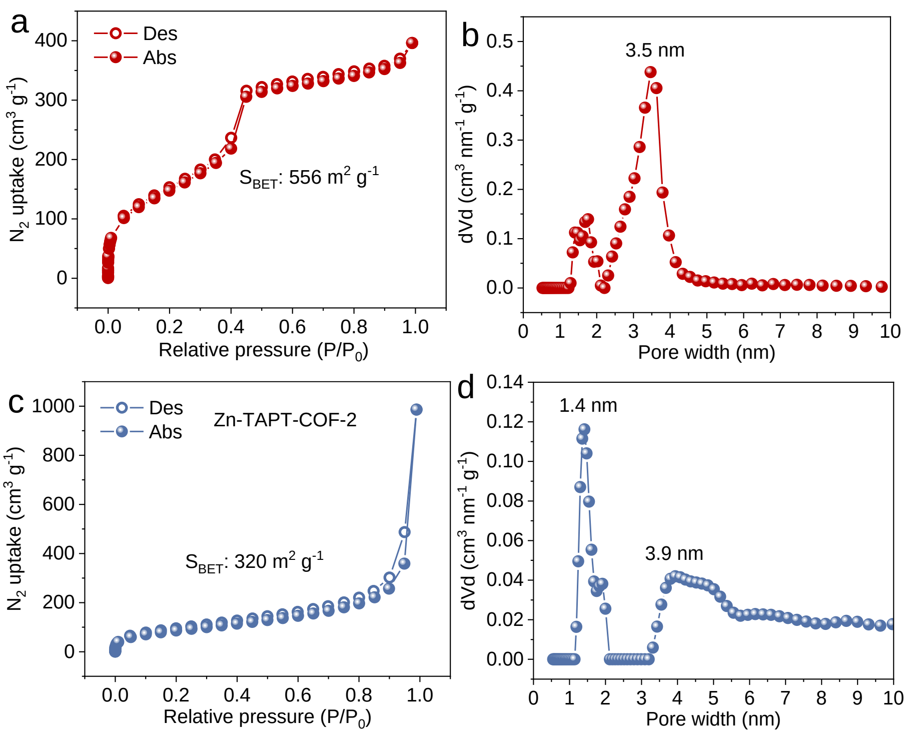

Figure S20. N_2 absorption and desorption isotherms (77 K), pore size distribution for (a, b) Zn-TAPT-COF-1 and (c, d) Zn-TAPT-COF-2.

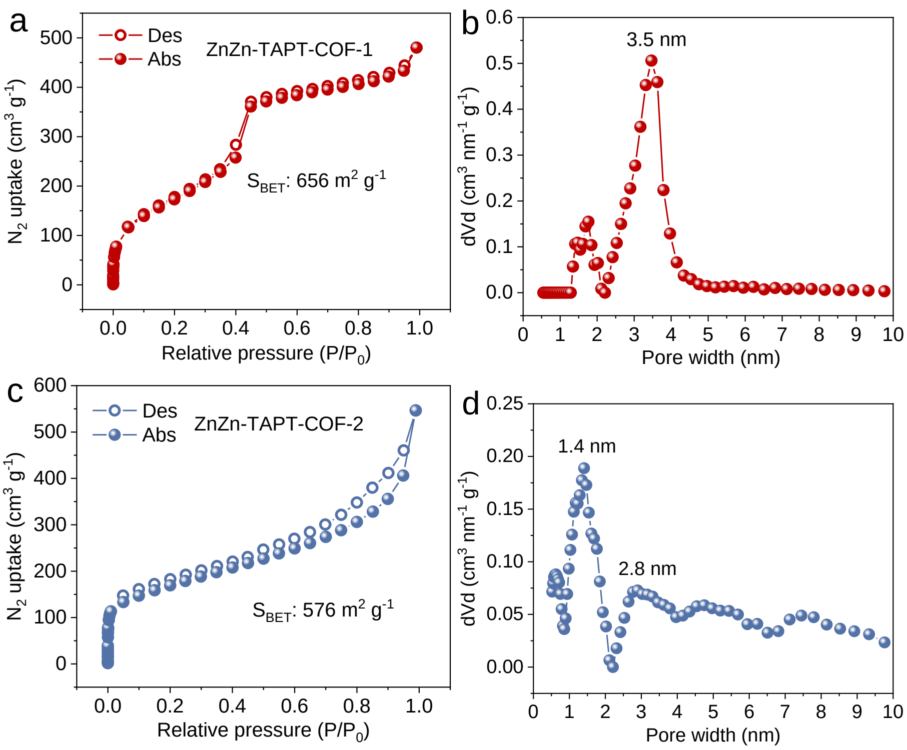

Figure S21. N_2 absorption and desorption isotherms (77 K), pore size distribution for (a, b) ZnZn-TAPT-COF-1 and (c, d) ZnZn-TAPT-COF-2.

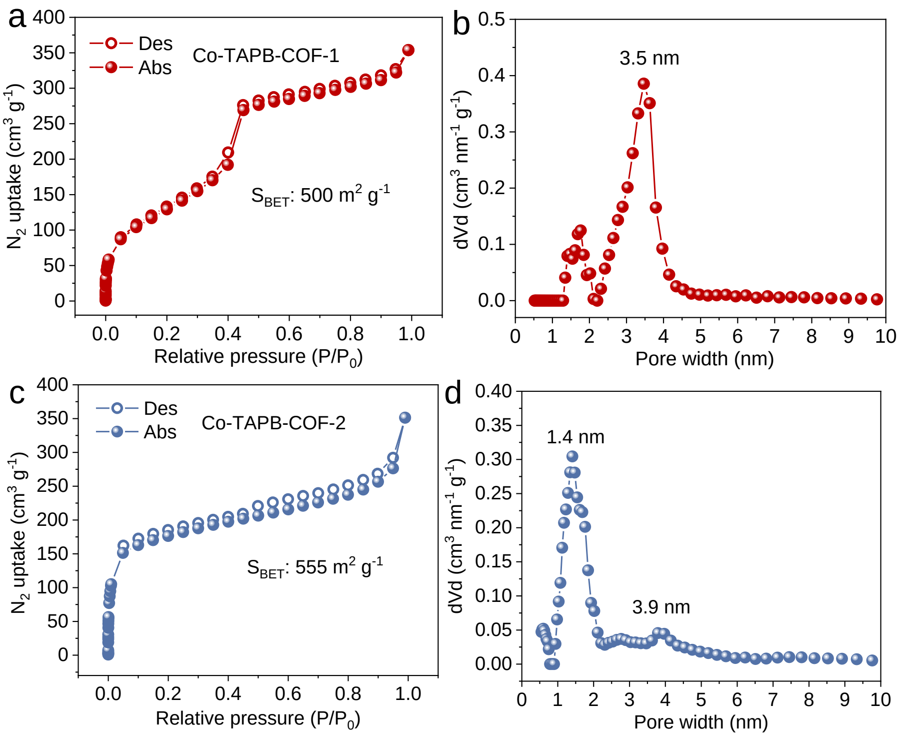

Figure S22. N_2 absorption and desorption isotherms (77 K), pore size distribution for (a, b) Co-TAPB-COF-1 and (c, d) Co-TAPB-COF-2.

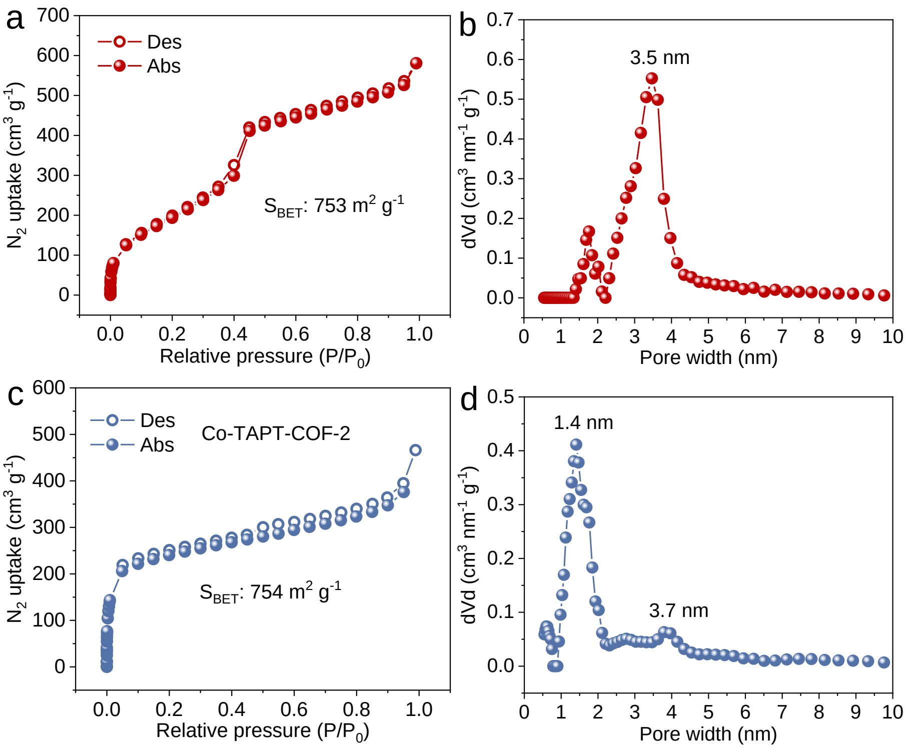

Figure S23. N_2 absorption and desorption isotherms (77 K), pore size distribution for (a, b) Co-TAPT-COF-1 and (c, d) Co-TAPT-COF-2.

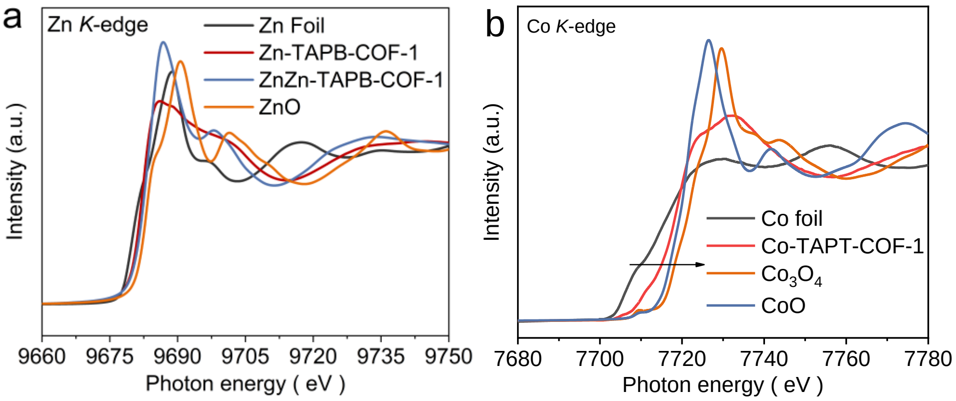

Figure S24. XANES spectra of (a) Zn foil, Zn-TAPB-COF-1, ZnZn-TAPB-COF-1, ZnO and (b) Co foil, Co-TAPT-COF-1, Co_3O_4, CoO respectively.

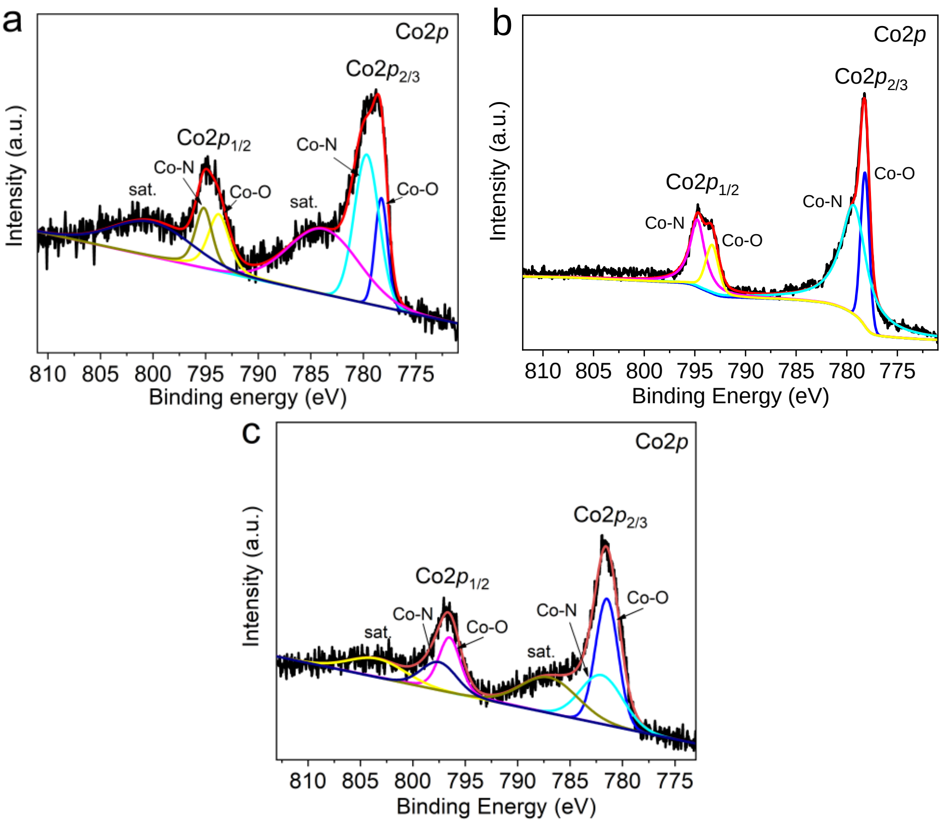

Figure S25. Co2*p* XPS spectra of (a) Co-TAPB-COF-1 and (b) Co-TAPT-COF-1 and (c) Co-TAPT-COF-2.

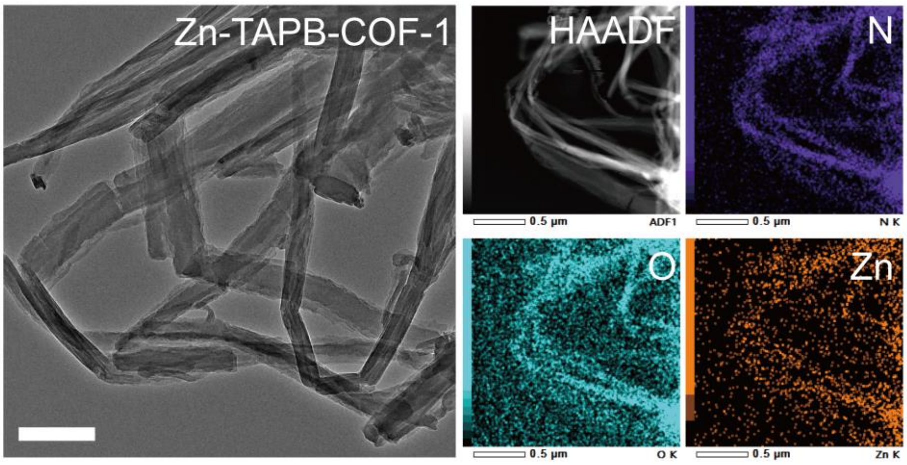

Figure S26. TEM images for Zn-TAPB-COF-1 (scale bar: 200 nm)

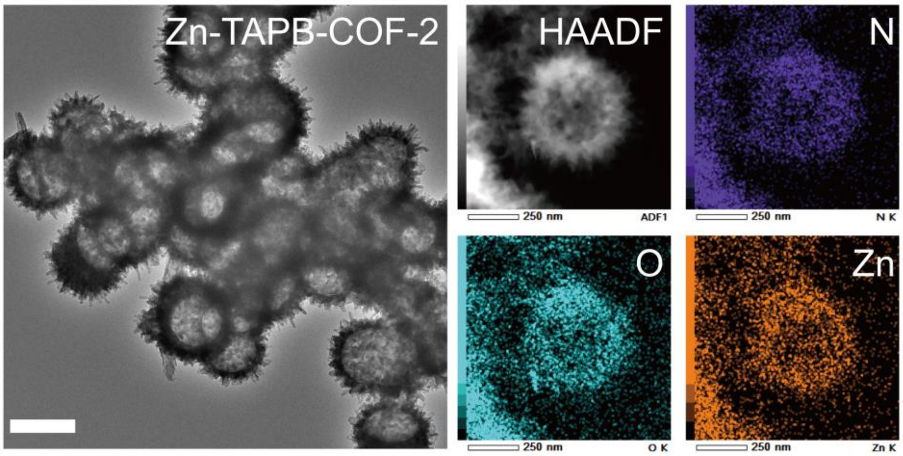

Figure S27. TEM images for Zn-TAPB-COF-2 (scale bar: 500 nm)

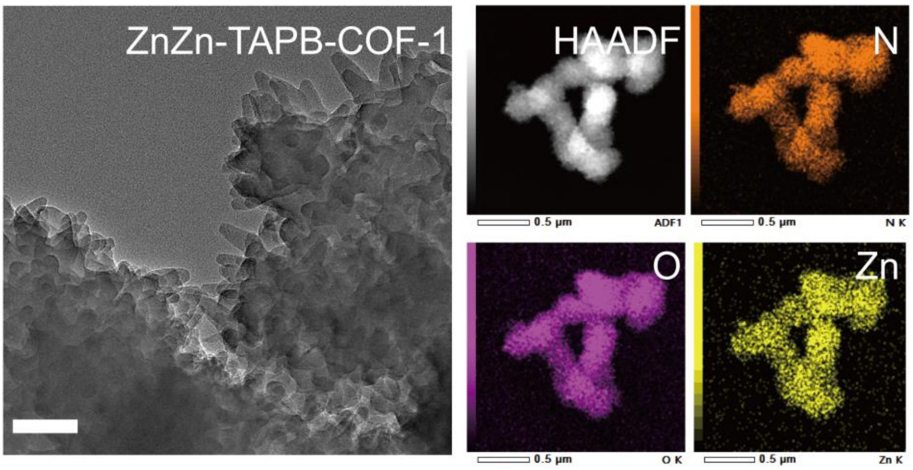

Figure S28. TEM images for ZnZn-TAPB-COF-1 (scale bar: 100 nm)

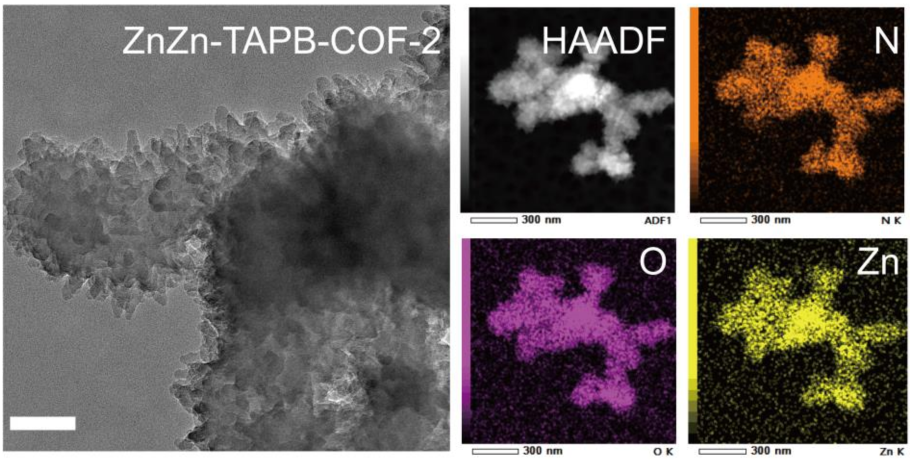

Figure S29. TEM images for ZnZn-TAPB-COF-2 (scale bar: 100 nm)

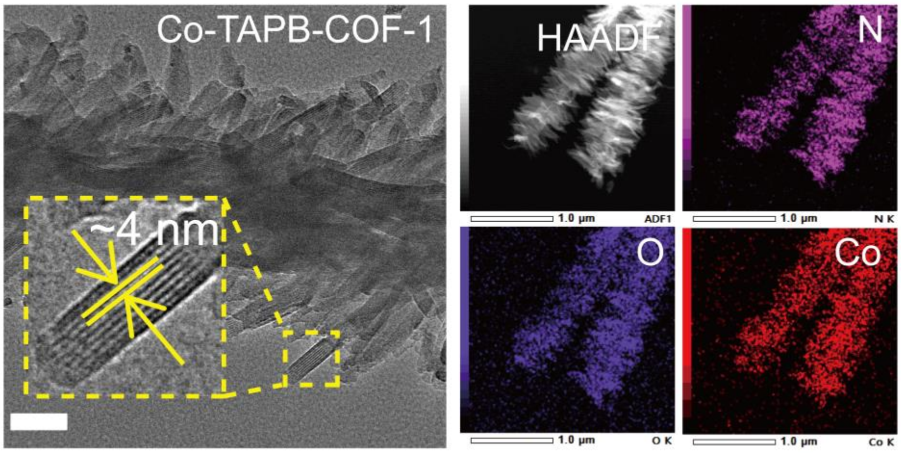

Figure S30. TEM images for Co-TAPB-COF-1 (scale bar: 100 nm)

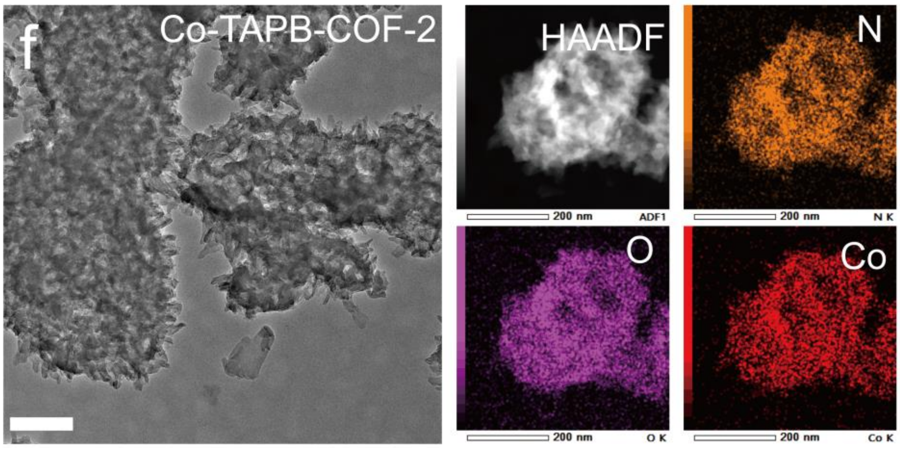

Figure S31. TEM images for Co-TAPB-COF-2 (scale bar: 200 nm)

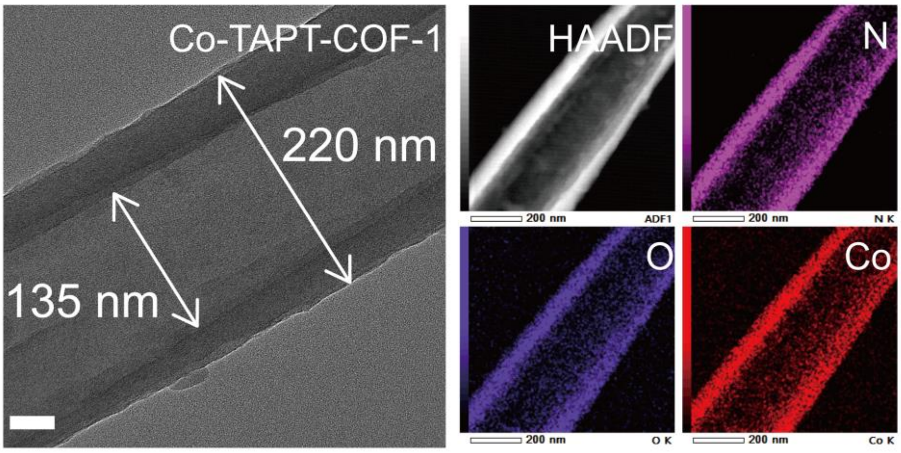

Figure S32. TEM images for Co-TAPT-COF-1 (scale bar: 50 nm)

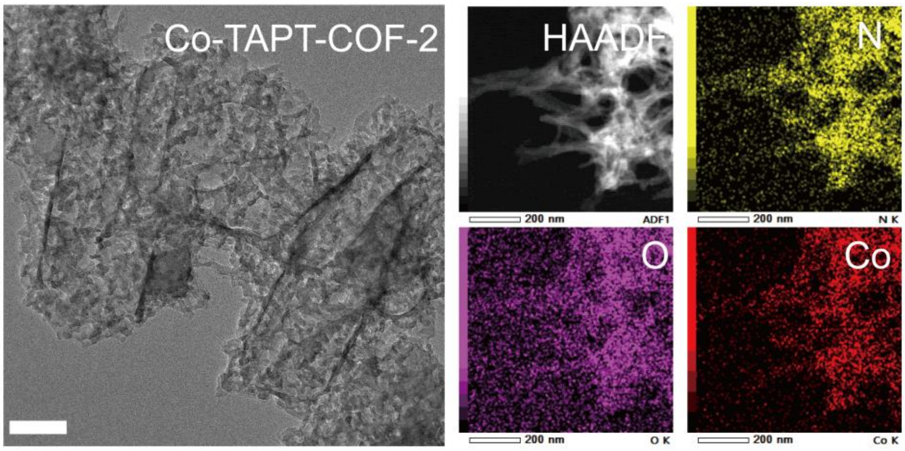

Figure S33. TEM images for Co-TAPT-COF-2 (scale bar: 100 nm)

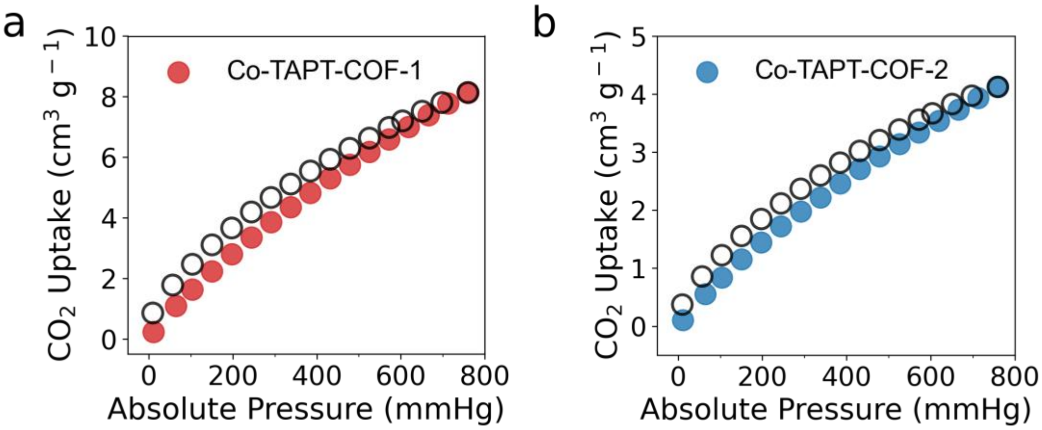

Figure S34. CO_2 adsorption (filled circles) and desorption (open circles) isotherms for (a) Co-TAPT-COF-1 and (b) Co-TAPT-COF-2 at 298 K.

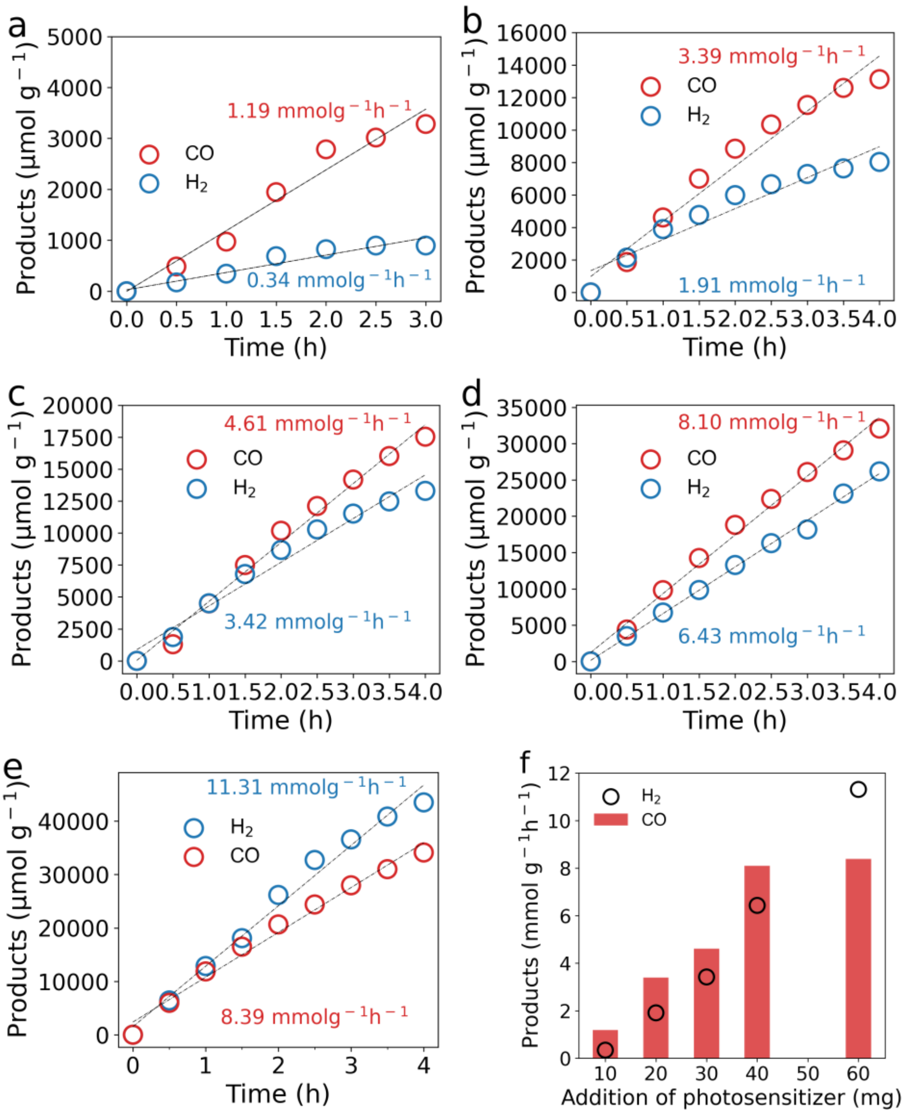

Figure S35. Time-dependent production rate of CO and H_2 with 10 mg Co-TAPT-COF-1 as catalyst in solvent system (H_2O/ TEOA / CH_3CN = 10 mL / 20 mL / 70 mL) with different dosages of $[Ru(bpy)_3]Cl_2{\cdot}6H_2O$: (a) 10 mg; (b) 20 mg; (c) 30 mg; (d) 40 mg; (e) 60 mg and (f) Production rate summary with different dosages of $[Ru(bpy)_3]Cl_2{\cdot}6H_2O$

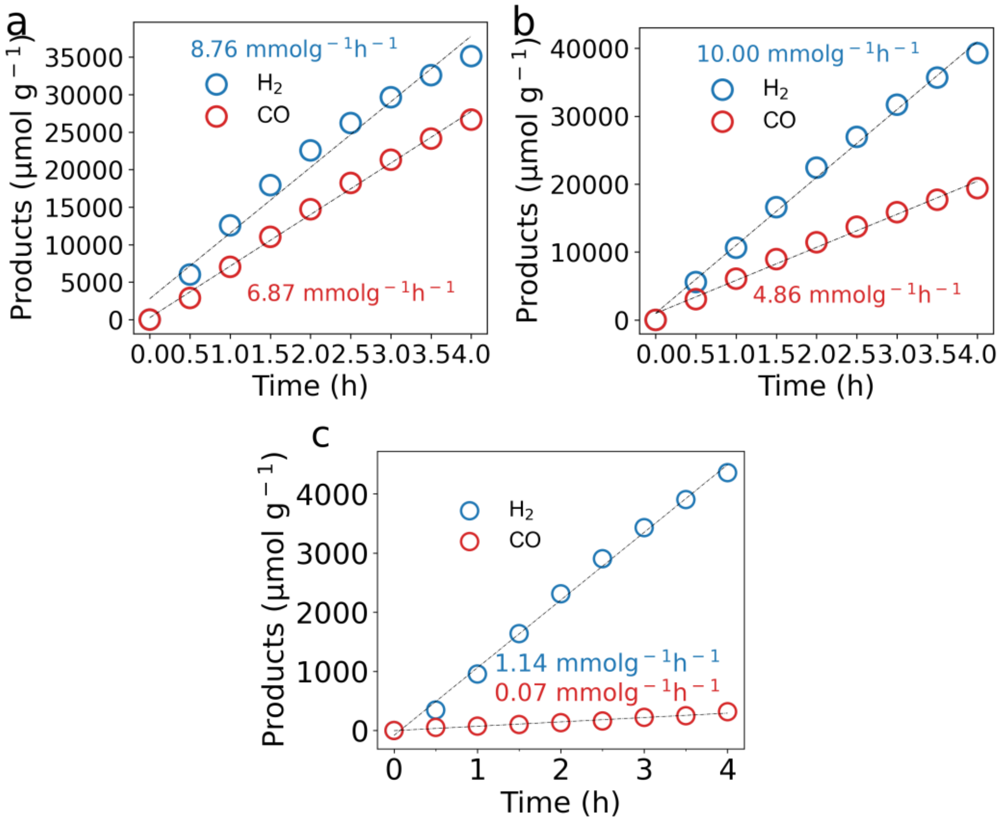

Figure S36. Time-dependent production rate of CO and H_2 in different solvent systems. (a) H_2O/ TEOA / CH_3CN = 10 mL / 30 mL / 60 mL with 10 mg Co-TAPT-COF-1 and 60 mg $[Ru(bpy)_3]Cl_2·6H_2O$, (b) H_2O/ TEOA / CH_3CN = 5 mL / 20 mL / 75 mL with 10 mg Co-TAPT-COF-1 and 60 mg $[Ru(bpy)_3]Cl_2·6H_2O$, (c) $KHCO_3$ aqueous solution (0.1 M) = 100 mL with 25 mg Co-TAPT-COF-1, 95 mg $[Ru(bpy)_3]Cl_2·6H_2O$ and 1.76 g ascorbic acid.

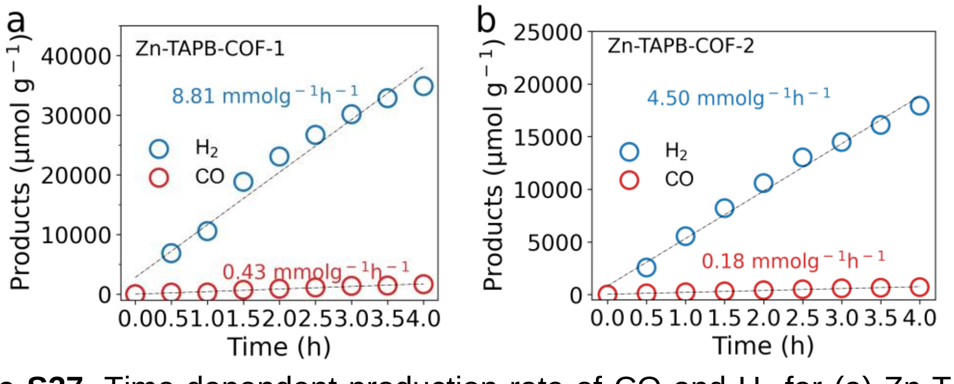

Figure S37. Time-dependent production rate of CO and H_2 for (a) Zn-TAPB-COF-1, (b) Zn-TAPB-COF-2.

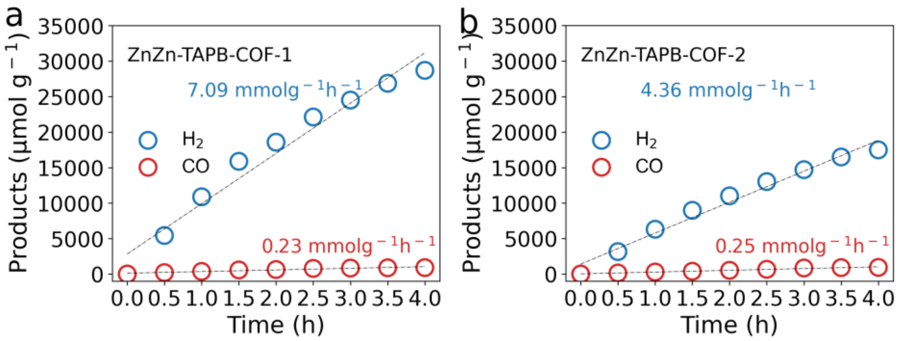

Figure S38. Time-dependent production rate of CO and H_2 for (a) ZnZn-TAPB-COF-1, (b) ZnZn-TAPB-COF-2.

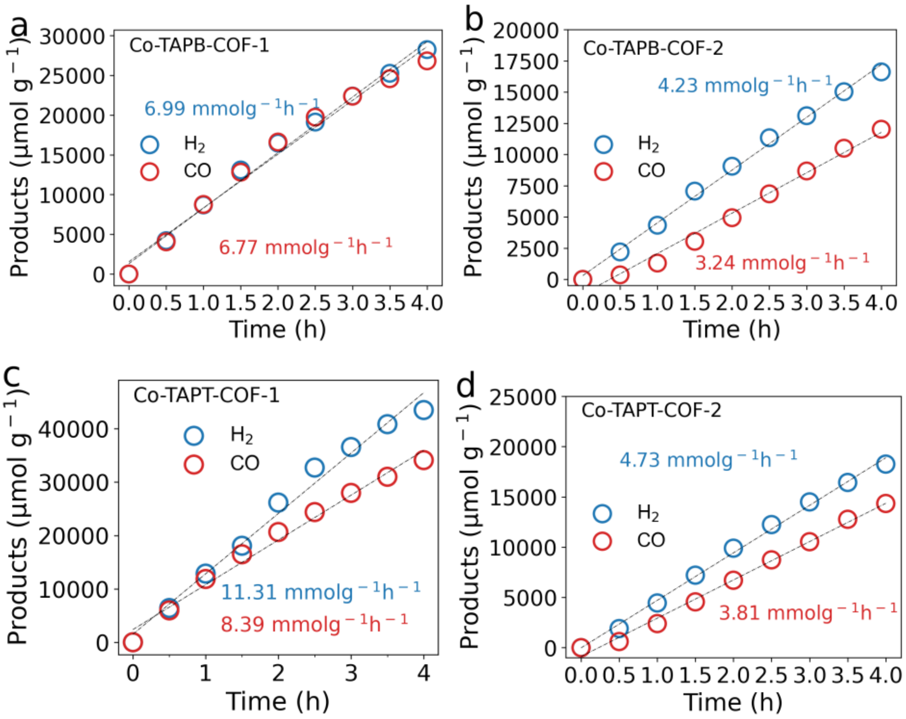

Figure S39. Time-dependent production rate of CO and H_2 with (a) Co-TAPB-COF-1, (b) Co-TAPB-COF-2, (c) Co-TAPT-COF-1 and (d) Co-TAPT-COF-2.

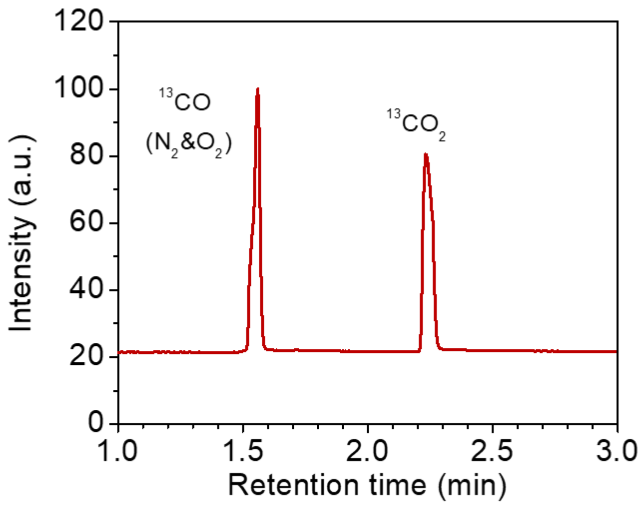

Figure S40. GC-MS spectrum for photoreduction $^{13}CO_2$ with Co-TAPT-COF-1 as photocatalyst.

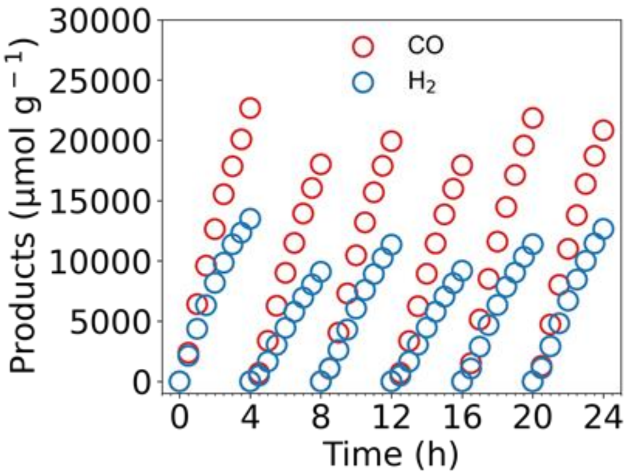

Figure S41. Stability test of Co-TAPT-COF-1 (stored for about one year) in five recycles for the photocatalytic CO_2 reduction reactions.

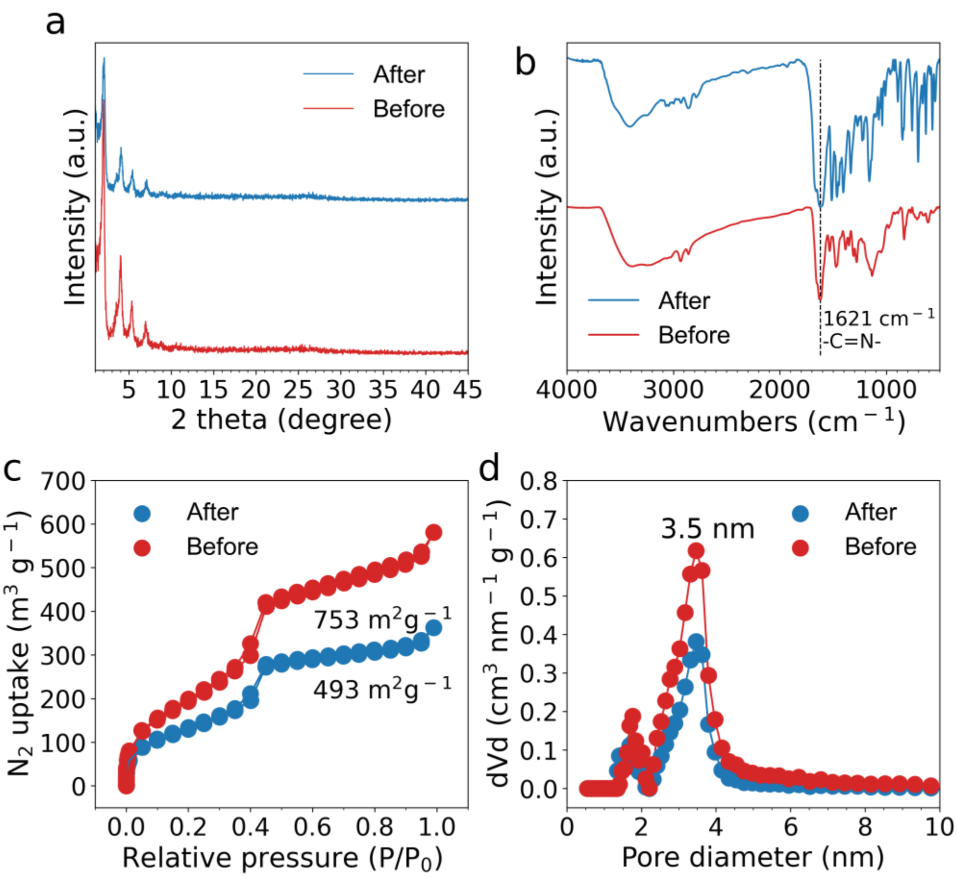

Figure S42. Post-catalysis characterizations of the Co-TAPT-COF-1. (a) PXRD patterns, (b) FT-IR spectra, (c) N_2 absorption- desorption isotherms (77 K) and (d) pore size distributions for Co-TAPT-COF-1 before and after photocatalysis.

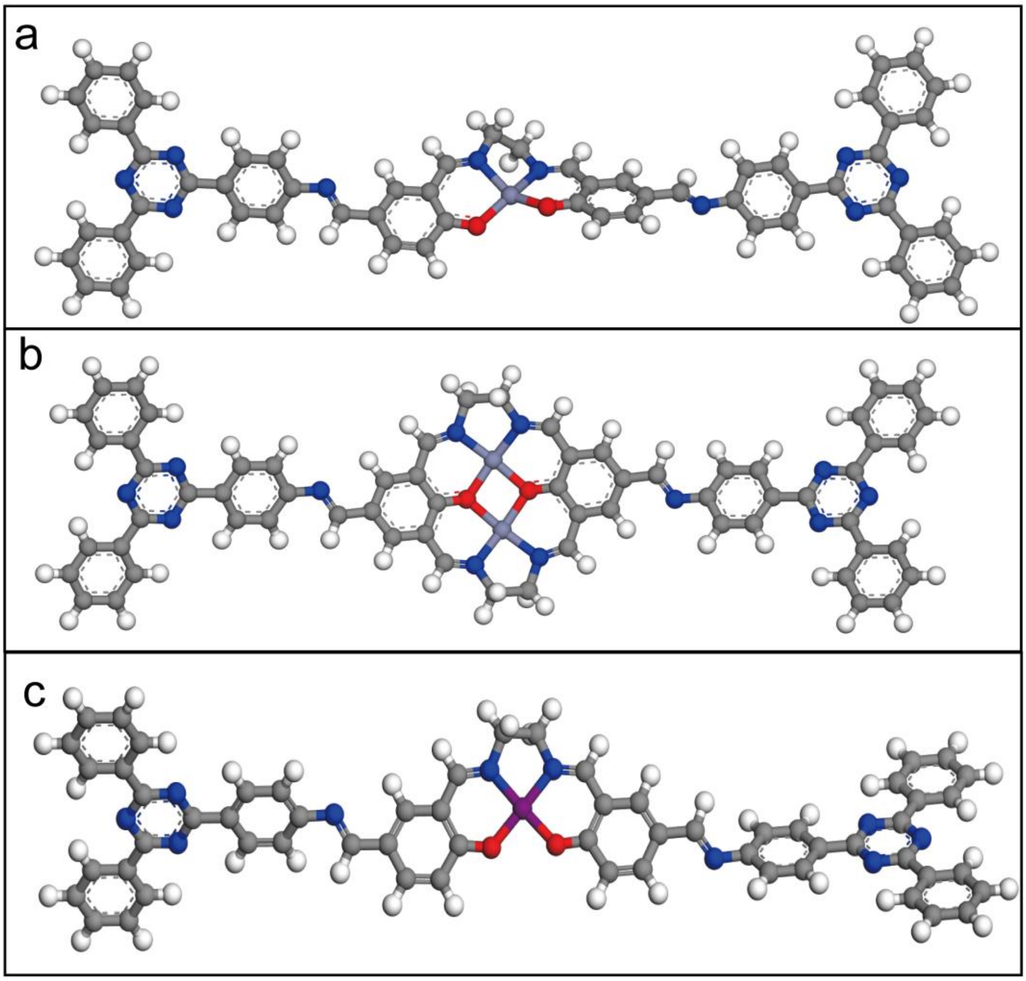

Figure S43. Calculation models for (a) Zn-TAPT-COF, (b) ZnZn-TAPT-COF and (c) Co-TAPT-COF, respectively.

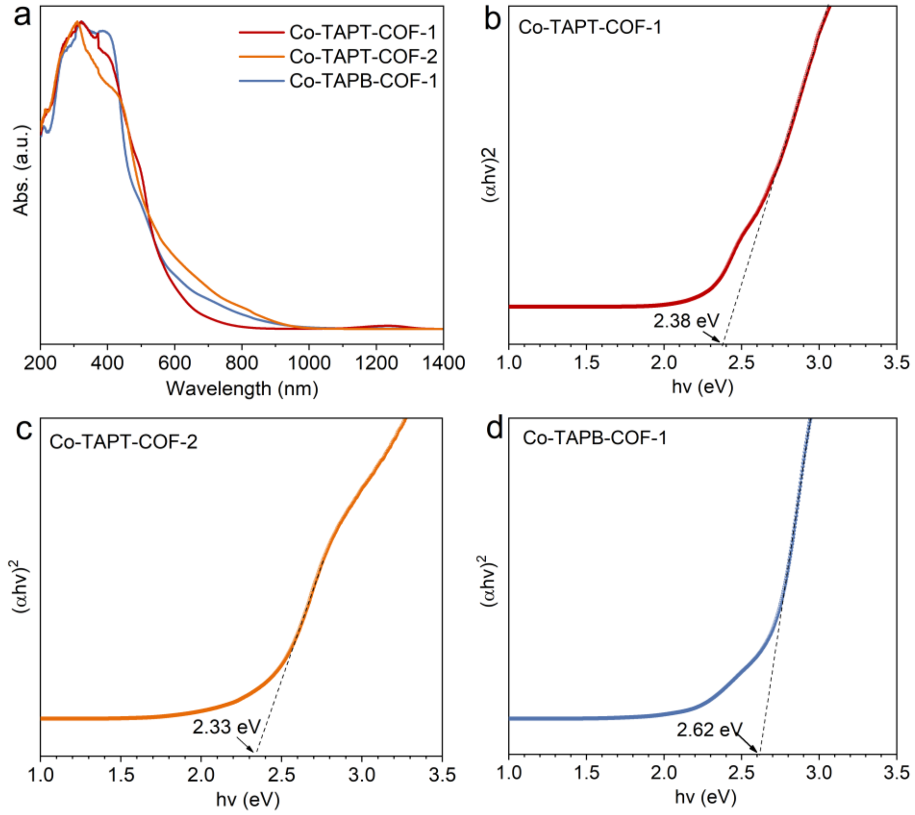

Figure S44. (a) Diffuse reflectance UV−visible spectra of Co(salen)-COFs; Tauc plots for (b) Co-TAPT-COF-1, (c) Co-TAPT-COF-2, (d) Co-TAPB-COF-1.

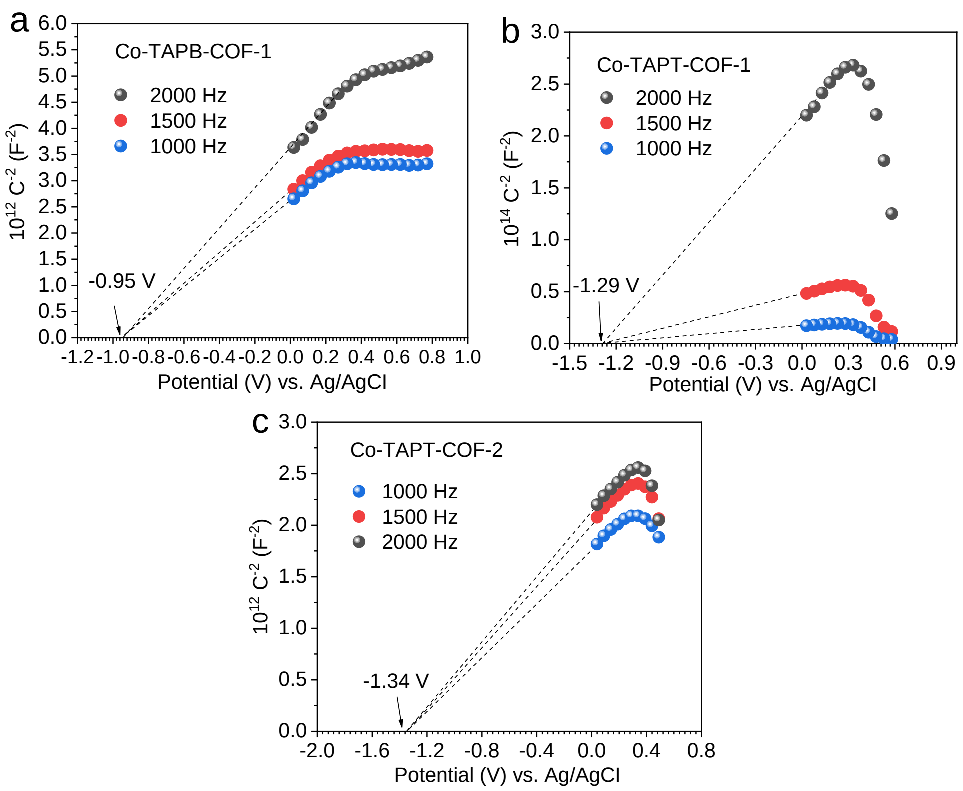

Figure S45. Mott-Schottky plots for (a) Co-TAPB-COF-1, (b) Co-TAPT-COF-1 and (c) Co-TAPT-COF-2.

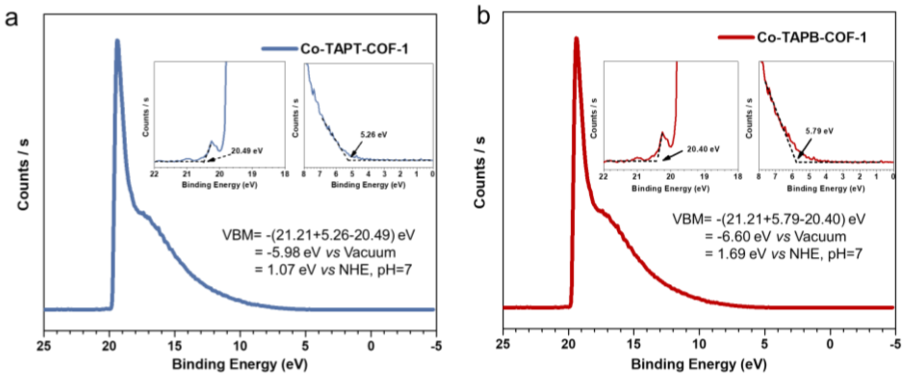

Figure S46. Ultraviolet photoelectron spectra for (a) Co-TAPB-COF-1 and (b) Co-TAPT-COF-1.

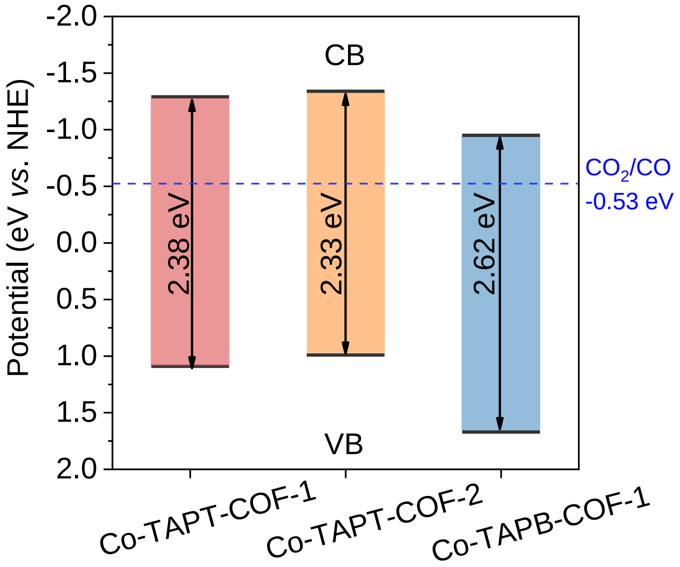

Figure S47. Band position diagram for Co-TAPT-COF-1, Co-TAPT-COF-2 and Co-TAPB-COF-1 (pH=7).

Table S1. Yields of M(salen)-COFs synthesized in different routes.

Samples	Zn-TAPB-COF		Zn-TAPT-COF		Co-TAPB-COF		ZnZn-TAPB-COF		ZnZn-TAPT-COF	
Synthetic route	One-step	Two-step	One-step	Two-step	One-step	Two-step	One-step	Two-step	One-step	Two-step
Yield (%)	87	67	67	66	83	53	81	39	75	36

Table S2. Fractional atomic coordinates for the unit cell of Zn-TAPB-COF-1.

**Zn-TAPB-COF-1**: Space group: *P1*<br>a=51.444(1) Å, b=51.235(8) Å, c=7.373(2) Å, α=74.6458°, β=98.9531°, γ=121.1356°							
**Atom**	**x**	**y**	**z**	**Atom**	**x**	**y**	**z**
C1	0.42564	0.00727	0.19833	C196	0.46634	0.01446	0.73679
C2	0.50437	0.07375	0.28579	C197	0.54298	0.08052	0.85096
C3	0.47228	0.05594	0.22359	C198	0.51105	0.06231	0.79107
C4	0.51527	0.1044	0.30664	C199	0.55257	0.1104	0.87914
O7	0.51702	0.03338	0.28796	O202	0.55785	0.04215	0.83705
C8	0.52476	0.06215	0.31803	C203	0.56445	0.07043	0.87178
C9	0.54568	0.12313	0.35922	C204	0.58279	0.13007	0.92845
N10	0.45844	0.02617	0.23007	N205	0.49847	0.03332	0.78276
C11	0.55491	0.08088	0.37023	C206	0.59461	0.09032	0.91944
C12	0.56535	0.11098	0.3921	C207	0.60378	0.11977	0.94901
C16	0.55693	0.15557	0.3732	C211	0.592	0.16144	0.95297
N17	0.58502	0.17281	0.42315	N212	0.61963	0.17983	1.00467
C19	0.59995	0.20505	0.43356	C214	0.63205	0.21154	1.02054
C20	0.62837	0.21758	0.52431	C215	0.66067	0.226	1.10671
C21	0.58804	0.22475	0.34739	C216	0.61796	0.22933	0.93747
C22	0.64469	0.24913	0.53223	C217	0.67572	0.25793	1.10441
C24	0.60434	0.25638	0.35472	C219	0.63314	0.26135	0.93246
C26	0.63283	0.26898	0.44826	C221	0.66254	0.27622	1.01171
C29	0.65048	0.30281	0.45295	C224	0.67991	0.31081	0.98854
C30	0.68224	0.31923	0.44269	C225	0.7114	0.3275	0.96343
N31	0.43421	0.96375	0.3552	N226	0.47474	0.9708	0.86968
C32	0.49805	0.93115	0.37034	C227	0.53617	0.93479	0.89544
C33	0.48631	0.90199	0.32894	C228	0.52301	0.90447	0.86747
O35	0.4926	0.97146	0.42678	O230	0.53358	0.97831	0.92832
C36	0.47916	0.94299	0.37506	C231	0.51855	0.94856	0.89011
C37	0.45518	0.88398	0.29231	C232	0.49167	0.88718	0.83464
C38	0.4133	0.97561	0.33995	C233	0.45401	0.98228	0.86836
C39	0.44754	0.92508	0.34098	C234	0.48679	0.93129	0.85747
C40	0.42569	0.9351	0.35475	C235	0.46567	0.94236	0.86338
C41	0.43588	0.89552	0.29973	C236	0.47381	0.90078	0.82926

C46	0.44357	0.85316	0.24943	C241	0.47783	0.85487	0.80974
N47	0.41513	0.83599	0.20547	N242	0.44876	0.83854	0.77813
C49	0.4016	0.80503	0.17236	C244	0.43245	0.80683	0.75569
C50	0.3729	0.79227	0.08507	C245	0.40369	0.79586	0.66897
C51	0.41511	0.7863	0.23775	C246	0.44392	0.7863	0.82283
C52	0.3575	0.76118	0.0663	C247	0.38695	0.76527	0.64176
C54	0.39965	0.75518	0.22154	C249	0.42725	0.75569	0.79619
C56	0.37028	0.7418	0.13933	C251	0.39879	0.74493	0.70189
C59	0.35283	0.70781	0.13444	C254	0.38184	0.71292	0.66332
C60	0.32087	0.69123	0.13676	C255	0.34999	0.69563	0.6679
Zn65	0.47606	0.99876	0.32541	Zn260	0.51662	1.0062	0.85487
C66	0.99872	0.42214	0.55884	C261	1.02736	0.42379	1.0918
C67	0.93251	0.43546	0.45336	C262	0.96155	0.43845	0.982
C68	0.94964	0.4225	0.52969	C263	0.97827	0.42489	1.05922
C69	0.89957	0.41665	0.49099	C264	0.92811	0.41982	1.00902
O72	0.97056	0.48516	0.2877	O267	1.00083	0.48847	0.83055
C73	0.9441	0.46745	0.33198	C268	0.97407	0.47095	0.86747
C74	0.88082	0.42791	0.41889	C269	0.90992	0.43187	0.93476
N75	0.9815	0.43841	0.50519	N270	1.01051	0.44063	1.04498
C76	0.92223	0.47826	0.25904	C271	0.95284	0.48253	0.79122
C77	0.89272	0.45983	0.29826	C272	0.92301	0.46439	0.82175
C81	0.84822	0.40805	0.46002	C276	0.87679	0.41245	0.96614
N82	0.82907	0.41705	0.37819	N277	0.85866	0.42348	0.89602
C84	0.79675	0.39988	0.40013	C279	0.82615	0.40727	0.91422
C85	0.7804	0.4147	0.31012	C280	0.81011	0.42166	0.80811
C86	0.78099	0.36892	0.50455	C281	0.80989	0.37771	1.03333
C87	0.74889	0.39926	0.32362	C282	0.77838	0.40668	0.81573
C89	0.74943	0.3534	0.51786	C284	0.77826	0.36275	1.04168
C91	0.73289	0.36829	0.4269	C286	0.76197	0.37681	0.93148
C94	0.69916	0.35137	0.43799	C289	0.72812	0.36012	0.93956
C95	0.6837	0.3674	0.43617	C290	0.71242	0.37631	0.9279
N96	0.04023	0.47367	0.41598	N291	0.06994	0.4764	0.97031
C97	0.06906	0.56741	0.04972	C292	0.09933	0.57035	0.60585
C98	0.09901	0.58641	0.02582	C293	0.12913	0.58881	0.57618
O100	0.02947	0.52059	0.19715	O295	0.05983	0.5237	0.75308
C101	0.05685	0.53618	0.17485	C296	0.08713	0.53902	0.72913
C102	0.12121	0.57677	0.12216	C297	0.15119	0.57858	0.6656
C103	0.03271	0.44586	0.56536	C298	0.06176	0.4466	1.10211
C104	0.07873	0.52582	0.26952	C299	0.10879	0.52798	0.81774
C105	0.07093	0.49769	0.38091	C300	0.10074	0.49966	0.92834
C106	0.11151	0.54835	0.23674	C301	0.14153	0.55009	0.77947
C111	0.15399	0.59872	0.08824	C306	0.1838	0.60039	0.62667
N112	0.174	0.59077	0.16837	N307	0.20422	0.59299	0.70589
C114	0.20644	0.60884	0.15119	C309	0.23651	0.61193	0.68249
C115	0.22331	0.59502	0.25001	C310	0.25414	0.59779	0.75312
C116	0.2218	0.63954	0.04295	C311	0.251	0.6439	0.59516
C117	0.25496	0.61132	0.24323	C312	0.28567	0.61488	0.73507

C119	0.25357	0.65596	0.036	C314	0.28259	0.66114	0.57852
C121	0.27051	0.64204	0.1364	C316	0.30028	0.64681	0.64805
C124	0.30425	0.65942	0.13185	C319	0.3339	0.66524	0.6353
C125	0.3199	0.64391	0.12729	C320	0.35009	0.65209	0.59813
Zn130	0.00533	0.48004	0.34894	Zn325	0.03515	0.48293	0.898
C131	0.57459	0.58179	-0.03956	C326	0.61548	0.59824	0.44572
C132	0.56929	0.50629	0.21939	C327	0.60399	0.51869	0.68523
C133	0.58141	0.53673	0.13965	C328	0.61758	0.54929	0.60505
C134	0.58913	0.49227	0.25039	C329	0.62238	0.50294	0.72534
O137	0.51791	0.49319	0.24894	O332	0.55353	0.50866	0.70481
C138	0.537	0.48514	0.27561	C333	0.57142	0.49907	0.73675
C139	0.57838	0.46172	0.32518	C334	0.6102	0.47239	0.80688
N140	0.56456	0.55262	0.10068	N335	0.60217	0.56674	0.56318
C141	0.52734	0.45232	0.36546	C336	0.55995	0.46609	0.83026
C142	0.54647	0.4413	0.38705	C337	0.57793	0.45358	0.86258
C146	0.59843	0.44846	0.34245	C341	0.62907	0.45749	0.83854
N147	0.58698	0.41855	0.36401	N342	0.61677	0.42728	0.88813
C149	0.60415	0.40311	0.37973	C344	0.63291	0.41053	0.91667
C150	0.58979	0.37401	0.33265	C345	0.61746	0.38026	0.88583
C151	0.6343	0.41563	0.44794	C346	0.66369	0.42373	0.97184
C152	0.60522	0.35759	0.35047	C347	0.63279	0.36389	0.89835
C154	0.64962	0.39897	0.47028	C349	0.67902	0.40727	0.98732
C156	0.63534	0.36954	0.42234	C351	0.66404	0.37726	0.94726
C159	0.65178	0.35176	0.44201	C354	0.68075	0.36016	0.95392
C160	0.63562	0.31914	0.45863	C355	0.66503	0.32731	0.98794
N161	0.52468	0.57331	0.01073	N356	0.56458	0.59055	0.46694
C162	0.42973	0.50567	0.17786	C357	0.46876	0.5246	0.62526
C163	0.41185	0.51761	0.17619	C358	0.45114	0.53695	0.61765
O165	0.47794	0.51393	0.15887	O360	0.51644	0.53188	0.60928
C166	0.4623	0.52608	0.13807	C361	0.50127	0.54453	0.58566
C167	0.42397	0.55182	0.13276	C362	0.46348	0.5712	0.56826
C168	0.55633	0.59712	-0.02647	C363	0.597	0.61355	0.45759
C169	0.47426	0.56037	0.07907	C364	0.51383	0.57889	0.52312
C170	0.50245	0.58149	0.01954	C365	0.54244	0.59943	0.46695
C171	0.45314	0.5717	0.08412	C366	0.49284	0.59082	0.5221
C176	0.40369	0.56365	0.14667	C371	0.44258	0.58276	0.57236
N177	0.41414	0.59353	0.11777	N372	0.45208	0.61242	0.54735
C179	0.39793	0.60893	0.12812	C374	0.43345	0.62548	0.55501
C180	0.41172	0.64105	0.05519	C375	0.44416	0.65713	0.47439
C181	0.36938	0.59333	0.21058	C376	0.40537	0.60787	0.64553
C182	0.39699	0.65745	0.05661	C377	0.42717	0.67111	0.4816
C184	0.35465	0.60976	0.21396	C379	0.38838	0.62175	0.65313
C186	0.36796	0.64203	0.13335	C381	0.39914	0.65363	0.57287
C189	0.35177	0.6594	0.13124	C384	0.38199	0.66875	0.595
C190	0.36792	0.69144	0.1332	C385	0.39753	0.69934	0.62445
Zn195	0.52074	0.53254	0.13223	Zn390	0.55853	0.54866	0.58637

Table S3. Fractional atomic coordinates for the unit cell of ZnZn-TAPB-COF-1.

ZnZn-TAPB-COF-1: Space group: *P1*

a=51.291(3) Å, b=51.194(2) Å c= 7.137(7) Å, α=88.8966°, β=92.1975°, γ=119.7291°

**Atom**	**x**	**y**	**z**	**Atom**	**x**	**y**	**z**
C1	0.41882	0.00344	0.2865	C229	0.45962	0.02809	0.79308
C2	0.49915	0.06795	0.28784	C230	0.53975	0.09254	0.79206
C3	0.46749	0.05086	0.28962	C231	0.50814	0.07546	0.79668
C4	0.51282	0.10116	0.28755	C232	0.55334	0.12572	0.79107
O7	0.50827	0.02771	0.28419	O235	0.54885	0.05225	0.78805
C8	0.51863	0.05582	0.28445	C236	0.55923	0.08039	0.78909
C9	0.54266	0.11922	0.28323	C237	0.58318	0.14389	0.78988
N10	0.45169	0.02037	0.29619	N238	0.49242	0.045	0.80155
C11	0.55178	0.07622	0.28006	C239	0.59239	0.10082	0.78695
C12	0.56262	0.10606	0.27905	C240	0.60322	0.13074	0.78715
C15	0.55554	0.15217	0.28322	C243	0.59572	0.17688	0.79037
N16	0.5845	0.16968	0.2864	N244	0.6246	0.19494	0.79315
C18	0.60064	0.20195	0.28728	C246	0.63997	0.22729	0.79044
C19	0.63161	0.21647	0.32238	C247	0.67056	0.2426	0.75382
C20	0.58683	0.21936	0.24975	C248	0.62559	0.24405	0.82014
C21	0.6486	0.24779	0.32658	C249	0.6864	0.27393	0.73867
C23	0.60383	0.25074	0.25146	C251	0.64168	0.27547	0.81363
C25	0.63507	0.26575	0.29375	C253	0.6724	0.29122	0.76951
C28	0.65321	0.29964	0.29406	C256	0.68965	0.32519	0.76415
C29	0.68466	0.31507	0.27781	C257	0.72133	0.34136	0.77616
N30	0.42884	0.96017	0.30565	N258	0.46924	0.98479	0.78815
C31	0.49545	0.92936	0.2916	C259	0.53527	0.95346	0.77023
C32	0.48303	0.89805	0.29871	C260	0.52252	0.922	0.76967
O33	0.48666	0.96964	0.29062	O261	0.52688	0.99401	0.77621
C34	0.47605	0.94151	0.29513	C262	0.51605	0.9658	0.77536
C35	0.4521	0.87854	0.31085	C263	0.49143	0.90257	0.77565
C36	0.40612	0.96931	0.32207	C264	0.44726	0.99408	0.82667
C37	0.44293	0.92106	0.30586	C265	0.48285	0.94546	0.78068
C38	0.42168	0.93087	0.31198	C266	0.46176	0.95542	0.78736
C39	0.43236	0.89004	0.31338	C267	0.47197	0.91439	0.78162
C44	0.44086	0.84587	0.32261	C272	0.47942	0.86964	0.77349
N45	0.4123	0.82697	0.3321	N273	0.45084	0.8513	0.79043
C47	0.39849	0.79477	0.3383	C275	0.4358	0.81901	0.789
C48	0.36862	0.77819	0.3907	C276	0.40697	0.80338	0.85912
C49	0.41323	0.77941	0.28271	C277	0.44834	0.80269	0.71378
C50	0.35351	0.74677	0.38855	C278	0.39125	0.772	0.8638
C52	0.39795	0.74805	0.27532	C280	0.43247	0.77119	0.71608
C54	0.36772	0.73105	0.32761	C282	0.40386	0.75521	0.79514
C57	0.35124	0.69735	0.31317	C285	0.38682	0.72138	0.79729
C58	0.31956	0.68067	0.29098	C286	0.35512	0.70549	0.79202

Zn61	0.46927	0.99452	0.29414	Zn289	0.50976	1.01906	0.78816
C62	0.4226	0.47067	0.28536	C290	0.4584	0.4943	0.7876
C63	0.4942	0.42368	0.30073	C291	0.53135	0.44923	0.79634
C64	0.44145	0.40867	0.29209	C292	0.47864	0.43339	0.79615
C65	0.42037	0.42238	0.28652	C293	0.45717	0.44655	0.79298
N66	0.47346	0.43136	0.29588	N294	0.51038	0.45648	0.79279
N67	0.43662	0.45533	0.28679	N295	0.4729	0.47945	0.78976
Zn68	0.48006	0.47247	0.28894	Zn296	0.51634	0.49731	0.78647
C77	0.99725	0.40907	0.26607	C305	1.03374	0.44575	0.72428
C78	0.93355	0.42638	0.28768	C306	0.97017	0.4605	0.80082
C79	0.95039	0.41141	0.27912	C307	0.98719	0.44585	0.79146
C80	0.90047	0.40734	0.29801	C308	0.93718	0.4411	0.79398
O83	0.97386	0.47523	0.27535	O311	1.01016	0.50964	0.80977
C84	0.94585	0.45795	0.28699	C312	0.98212	0.49201	0.80979
C85	0.88253	0.41943	0.30349	C313	0.91889	0.45271	0.79759
N86	0.98066	0.42552	0.26342	N314	1.01753	0.46036	0.78894
C87	0.92572	0.47115	0.29949	C315	0.96157	0.50476	0.81639
C88	0.89588	0.45257	0.30513	C316	0.9317	0.48575	0.81071
C91	0.84958	0.39948	0.30659	C319	0.88616	0.4321	0.78778
N92	0.83156	0.41054	0.30123	N320	0.86746	0.44214	0.79774
C94	0.79923	0.39372	0.29406	C322	0.83527	0.42403	0.79354
C95	0.78357	0.4091	0.32143	C323	0.81833	0.43831	0.76386
C96	0.78284	0.36291	0.25059	C324	0.82025	0.39277	0.82262
C97	0.75218	0.39397	0.31469	C325	0.787	0.4219	0.76209
C99	0.75151	0.3481	0.23308	C327	0.78893	0.3763	0.82126
C101	0.73534	0.3631	0.2687	C329	0.77146	0.39049	0.79376
C104	0.70144	0.3467	0.26509	C332	0.73757	0.37297	0.79245
C105	0.68553	0.36259	0.25379	C333	0.72106	0.38794	0.80959
N106	0.04071	0.46182	0.23752	N334	0.07713	0.49712	0.7538
C107	0.07226	0.55936	0.25029	C335	0.10792	0.59427	0.76375
C108	0.10355	0.57783	0.25133	C336	0.13928	0.61276	0.75612
O109	0.03178	0.51072	0.24767	O337	0.06776	0.54559	0.77608
C110	0.05994	0.52784	0.24624	C338	0.09575	0.56277	0.76371
C111	0.12304	0.5661	0.24975	C339	0.15865	0.60084	0.7492
C112	0.03116	0.42968	0.22787	C340	0.06785	0.46518	0.76219
C113	0.08025	0.51472	0.24277	C341	0.11605	0.54973	0.75193
C114	0.07013	0.48355	0.23857	C342	0.10631	0.51876	0.74801
C115	0.11141	0.53483	0.24501	C343	0.14694	0.56965	0.74693
C120	0.15576	0.5875	0.25498	C348	0.19147	0.62133	0.74704
N121	0.17518	0.57821	0.2597	N349	0.20966	0.61048	0.75156
C123	0.20733	0.59718	0.26433	C351	0.24186	0.62731	0.75338
C124	0.22496	0.5847	0.32542	C352	0.25769	0.61268	0.81047
C125	0.22166	0.62742	0.20589	C353	0.2579	0.65758	0.69862
C126	0.25626	0.60197	0.33163	C354	0.28904	0.6281	0.81883
C128	0.25305	0.64457	0.20736	C356	0.28925	0.67296	0.70463
C130	0.27102	0.63215	0.27005	C358	0.30553	0.65863	0.76639
C133	0.30479	0.65028	0.27535	C361	0.33929	0.67518	0.77585

C134	0.32192	0.63564	0.26957	C362	0.3553	0.65945	0.76352
Zn137	0.00671	0.46868	0.25544	Zn365	0.04311	0.50356	0.78327
C138	0.5288	0.94769	0.28327	C366	0.56872	0.97179	0.76591
C139	0.57392	0.06545	0.27731	C367	0.61448	0.08988	0.78724
C140	0.58956	0.02818	0.28648	C368	0.62998	0.05236	0.79094
C141	0.57637	0.99375	0.27032	C369	0.61665	1.01783	0.77346
N142	0.5666	0.03724	0.27682	N370	0.60709	0.0616	0.7839
N143	0.54366	0.97693	0.28248	N371	0.58377	1.00107	0.77208
Zn144	0.52588	0.00283	0.28338	Zn372	0.56624	0.02719	0.77987
C153	0.58676	0.578	0.24289	C381	0.62289	0.60467	0.75383
C154	0.57133	0.49812	0.29107	C382	0.60783	0.5243	0.78153
C155	0.58593	0.52977	0.28409	C383	0.62208	0.556	0.77221
C156	0.59071	0.48428	0.291	C384	0.62748	0.51067	0.78524
O159	0.52229	0.48949	0.29189	O387	0.55859	0.51502	0.78177
C160	0.53987	0.47883	0.29411	C388	0.57644	0.50469	0.7855
C161	0.57907	0.4544	0.29431	C389	0.61605	0.48078	0.79225
N162	0.57145	0.54584	0.28277	N390	0.60733	0.57186	0.7702
C163	0.52709	0.44555	0.29863	C391	0.56403	0.47147	0.79336
C164	0.54603	0.43446	0.29949	C392	0.58314	0.46067	0.79691
C167	0.59951	0.44187	0.28998	C395	0.63639	0.46813	0.79248
N168	0.589	0.41299	0.29039	N396	0.62564	0.43918	0.79672
C170	0.60633	0.39794	0.28609	C398	0.64249	0.42364	0.79288
C171	0.59121	0.36721	0.24634	C399	0.62727	0.39259	0.8226
C172	0.63754	0.41265	0.32299	C400	0.67317	0.43809	0.75307
C173	0.60679	0.35135	0.2398	C401	0.64241	0.37623	0.82035
C175	0.653	0.39658	0.32327	C403	0.68814	0.42166	0.74532
C177	0.63794	0.36558	0.28187	C405	0.67322	0.39046	0.78199
C180	0.65442	0.34819	0.2814	C408	0.68975	0.37314	0.77965
C181	0.63899	0.31691	0.30978	C409	0.67452	0.34183	0.75131
N182	0.53483	0.56964	0.26895	N410	0.56997	0.5948	0.77447
C183	0.43788	0.50425	0.28465	C411	0.47313	0.5278	0.78338
C184	0.41946	0.51735	0.28728	C412	0.45418	0.54028	0.78144
O185	0.48642	0.51208	0.28089	O413	0.52206	0.53668	0.78195
C186	0.46929	0.52324	0.28142	C414	0.50449	0.54731	0.78103
C187	0.43127	0.5486	0.2873	C415	0.46536	0.57139	0.77692
C188	0.56677	0.59203	0.27801	C416	0.60182	0.61783	0.78355
C189	0.48232	0.55663	0.27934	C417	0.51693	0.5806	0.77764
C190	0.51326	0.57752	0.27469	C418	0.54784	0.60199	0.77731
C191	0.4624	0.56788	0.28306	C419	0.49643	0.59121	0.77588
C196	0.41049	0.56077	0.29272	C424	0.44388	0.58275	0.77363
N197	0.42069	0.58962	0.29355	N425	0.45313	0.61128	0.75421
C199	0.40301	0.60425	0.30082	C427	0.43489	0.62541	0.75188
C200	0.41617	0.63441	0.24669	C428	0.44779	0.65534	0.69321
C201	0.37342	0.58967	0.36376	C429	0.40518	0.6108	0.81272
C202	0.39973	0.64946	0.24483	C430	0.43166	0.67071	0.69621
C204	0.35713	0.6049	0.36596	C432	0.38916	0.62627	0.81874
C206	0.36978	0.63484	0.30234	C434	0.40227	0.6567	0.76333

C209	0.35205	0.65084	0.2974	C437	0.38565	0.67381	0.7771
C210	0.36655	0.68216	0.32321	C438	0.40139	0.70542	0.79883
Zn213	0.52838	0.52884	0.28147	Zn441	0.56413	0.55418	0.77703
C214	0.05428	0.57481	0.26233	C442	0.08962	0.60952	0.77716
C215	0.93688	0.5041	0.30726	C443	0.97232	0.53766	0.82827
C216	0.97492	0.55659	0.30308	C444	1.00963	0.59058	0.83177
C217	0.00935	0.57614	0.33072	C445	0.04418	0.6116	0.82357
N218	0.96512	0.52444	0.30343	N446	1.0005	0.55851	0.82688
N219	0.02521	0.56077	0.27249	N447	0.06057	0.59542	0.79445
Zn220	0.99903	0.51741	0.27364	Zn448	1.0348	0.55193	0.80193

Table S4. Fractional atomic coordinates for the unit cell of Co-TAPT-COF-1.

Co-TAPT-COF-1: Space group: *P1*

a=50.573(2) Å, b=50.127(7) Å, c=7.087(5) Å, α=74.4833°, β=97.2283°, γ=120.9587°.

Atom	x	y	z	Atom	x	y	z
C1	0.41406	0.00689	0.17893	C196	0.45916	0.01276	0.7206
C2	0.49367	0.07385	0.24481	C197	0.53713	0.07871	0.81097
C3	0.4615	0.05621	0.1849	C198	0.50516	0.0605	0.75478
C4	0.506	0.1052	0.26715	C199	0.54871	0.10984	0.83392
O7	0.50224	0.03097	0.23334	O202	0.54678	0.0374	0.78353
C8	0.51266	0.06047	0.27153	C203	0.5568	0.06649	0.82669
C9	0.53712	0.12308	0.31576	C204	0.57996	0.12872	0.87381
N10	0.44753	0.02562	0.19637	N205	0.4922	0.03055	0.75077
C11	0.54365	0.07827	0.31757	C206	0.58801	0.08526	0.86478
C12	0.55588	0.10932	0.34089	C207	0.59958	0.1161	0.89033
C16	0.54972	0.15607	0.33649	C211	0.5917	0.16158	0.89324
N17	0.57836	0.17278	0.38392	N212	0.62068	0.17964	0.92725
C19	0.59414	0.20516	0.40605	C214	0.63584	0.21229	0.94277
C20	0.62604	0.22014	0.43215	C215	0.66781	0.22798	0.96356
C21	0.57903	0.22235	0.40074	C216	0.62026	0.22918	0.93325
C22	0.64297	0.25203	0.44682	C217	0.68435	0.26011	0.97145
C24	0.59587	0.25422	0.41612	C219	0.63678	0.26142	0.94166
C26	0.62809	0.26957	0.43749	C221	0.66906	0.27731	0.95949
C29	0.64606	0.30368	0.44822	C224	0.68693	0.31169	0.96277
C30	0.67726	0.31894	0.45501	C225	0.71815	0.32709	0.96432
N31	0.42453	0.9661	0.3693	N226	0.46983	0.97181	0.87907
C32	0.48943	0.93588	0.36172	C227	0.53303	0.9394	0.88068
C33	0.47839	0.90547	0.33124	C228	0.52159	0.90881	0.85112
O35	0.48078	0.97579	0.41644	O230	0.52488	0.98022	0.92759
C36	0.4691	0.9465	0.37191	C231	0.5132	0.95109	0.88036
C37	0.44669	0.88524	0.31135	C232	0.48991	0.8893	0.8248
C38	0.40252	0.97731	0.34791	C233	0.44733	0.98182	0.87277
C39	0.43733	0.92636	0.35695	C234	0.48171	0.93191	0.8547
C40	0.41549	0.9363	0.38063	C235	0.46041	0.94254	0.87313
C41	0.42619	0.89573	0.32633	C236	0.47004	0.901	0.82765
C46	0.43557	0.85345	0.27529	C241	0.47804	0.85697	0.79508
N47	0.40663	0.8352	0.2408	N242	0.44872	0.83846	0.77134
C49	0.39236	0.80362	0.20703	C244	0.43374	0.80665	0.74026
C50	0.36049	0.78765	0.17986	C245	0.40195	0.79138	0.7082
C51	0.40869	0.78781	0.20678	C246	0.44953	0.79021	0.74345
C52	0.34485	0.75633	0.15538	C247	0.38595	0.76021	0.68008
C54	0.39308	0.75645	0.18242	C249	0.43367	0.75921	0.7138
C56	0.36095	0.74027	0.15733	C251	0.4017	0.74382	0.68159
C59	0.34425	0.70669	0.13678	C254	0.38515	0.7109	0.64991
C60	0.31302	0.69038	0.12969	C255	0.35394	0.69431	0.64245

Zn65	0.46437	0.99971	0.30418	Zn260	0.50817	1.00496	0.83502
C66	0.99362	0.4122	0.60897	C261	1.03302	0.41663	1.13916
C67	0.92789	0.42771	0.49796	C262	0.9694	0.43402	1.02762
C68	0.94596	0.41341	0.56838	C263	0.98651	0.41928	1.09858
C69	0.89577	0.41002	0.52635	C264	0.93539	0.4155	1.05427
O72	0.97066	0.47416	0.37311	O267	1.01244	0.48262	0.89682
C73	0.94184	0.45978	0.39676	C268	0.98355	0.46595	0.92706
C74	0.87783	0.4245	0.45392	C269	0.91834	0.42943	0.9827
N75	0.97733	0.42982	0.54254	N270	1.01758	0.43527	1.07626
C76	0.92453	0.47469	0.3243	C271	0.96449	0.48085	0.85078
C77	0.89242	0.45702	0.35281	C272	0.93378	0.46372	0.87621
C81	0.84406	0.40489	0.4826	C276	0.88451	0.41033	1.00871
N82	0.82635	0.41718	0.42462	N277	0.86739	0.42315	0.94253
C84	0.79327	0.39955	0.44126	C279	0.83428	0.4063	0.95321
C85	0.77789	0.41641	0.43053	C280	0.81938	0.42386	0.93453
C86	0.77586	0.36625	0.45862	C281	0.8164	0.37302	0.97199
C87	0.74566	0.40045	0.43991	C282	0.78713	0.40853	0.93909
C89	0.74366	0.35025	0.46748	C284	0.78423	0.35767	0.97552
C91	0.72814	0.36716	0.45752	C286	0.76917	0.3752	0.95961
C94	0.69391	0.35031	0.45903	C289	0.73495	0.35866	0.96191
C95	0.67901	0.36642	0.45661	C290	0.72016	0.37488	0.95911
N96	0.0335	0.46604	0.46478	N291	0.07381	0.47009	1.00606
C97	0.05859	0.55944	0.14438	C292	0.10083	0.56363	0.67733
C98	0.08981	0.58178	0.09813	C293	0.13189	0.58502	0.62404
O100	0.02097	0.50867	0.29424	O295	0.06295	0.51377	0.83291
C101	0.04935	0.528	0.25008	C296	0.0912	0.53225	0.78487
C102	0.11214	0.57266	0.15574	C297	0.15366	0.57505	0.677
C103	0.02777	0.43589	0.59835	C298	0.06724	0.43943	1.1367
C104	0.07147	0.51879	0.30657	C299	0.1126	0.52201	0.83789
C105	0.06314	0.48824	0.41328	C300	0.10355	0.49136	0.94875
C106	0.10271	0.54101	0.25925	C301	0.1438	0.54339	0.78338
C111	0.14518	0.5961	0.11152	C306	0.18659	0.59807	0.6271
N112	0.16533	0.58674	0.155	N307	0.20683	0.58881	0.66858
C114	0.19819	0.60618	0.13186	C309	0.2396	0.60885	0.64053
C115	0.21497	0.59077	0.14248	C310	0.25686	0.59421	0.64283
C116	0.21407	0.63945	0.11354	C311	0.25501	0.642	0.62699
C117	0.24717	0.60805	0.1342	C312	0.28905	0.61213	0.63152
C119	0.24636	0.65683	0.10668	C314	0.28726	0.65999	0.61726
C121	0.26329	0.64128	0.11805	C316	0.30467	0.64519	0.62107
C124	0.29752	0.65935	0.11836	C319	0.33887	0.6638	0.62044
C125	0.31357	0.64469	0.11339	C320	0.35536	0.65	0.60538
Zn130	0.00069	0.47077	0.41475	Zn325	0.04157	0.47616	0.95059
C131	0.58177	0.59397	-0.04903	C326	0.62909	0.60637	0.43243
C132	0.57144	0.51489	0.20529	C327	0.61648	0.52565	0.6797
C133	0.58479	0.5462	0.13135	C328	0.63032	0.55692	0.6003
C134	0.59041	0.4993	0.24757	C329	0.63471	0.50923	0.72652

O137	0.52029	0.50508	0.20346	O332	0.566	0.51724	0.67854
C138	0.53796	0.49467	0.24187	C333	0.58311	0.50622	0.72024
C139	0.57749	0.46749	0.31839	C334	0.62117	0.47753	0.80567
N140	0.56739	0.56209	0.08028	N335	0.61347	0.57347	0.54827
C141	0.52542	0.46015	0.32648	C336	0.56988	0.47191	0.81319
C142	0.54379	0.4474	0.36159	C337	0.58759	0.45844	0.85269
C146	0.59707	0.45279	0.34664	C341	0.63988	0.46185	0.83961
N147	0.58496	0.42185	0.38963	N342	0.6272	0.43093	0.9006
C149	0.6015	0.40493	0.41114	C344	0.6431	0.4133	0.92872
C150	0.58464	0.37242	0.4111	C345	0.62595	0.38079	0.92876
C151	0.63372	0.41967	0.43198	C346	0.67523	0.4277	0.95216
C152	0.59951	0.35489	0.42597	C347	0.64064	0.36314	0.94129
C154	0.64867	0.40224	0.44658	C349	0.68997	0.41017	0.96452
C156	0.63178	0.36969	0.44214	C351	0.67294	0.37784	0.95539
C159	0.6478	0.3515	0.45102	C354	0.68888	0.35978	0.95922
C160	0.63149	0.32014	0.44627	C355	0.67242	0.3282	0.96008
N161	0.53027	0.58333	-0.00529	N356	0.5765	0.59514	0.44902
C162	0.43469	0.51343	0.13856	C357	0.48117	0.52483	0.60454
C163	0.4152	0.52419	0.13621	C358	0.46143	0.53525	0.60083
O165	0.48524	0.52509	0.12044	O360	0.53173	0.53717	0.58442
C166	0.46798	0.53581	0.10419	C361	0.51421	0.54754	0.56403
C167	0.42628	0.55897	0.09813	C362	0.47202	0.56997	0.55527
C168	0.56191	0.60875	-0.05548	C363	0.60864	0.62072	0.42033
C169	0.47887	0.57033	0.05392	C364	0.52465	0.58192	0.50367
C170	0.50781	0.59216	0.00035	C365	0.55352	0.60375	0.44574
C171	0.45629	0.58057	0.05765	C366	0.50176	0.59188	0.50636
C176	0.40402	0.56942	0.10732	C371	0.44937	0.57971	0.56815
N177	0.41349	0.59956	0.08814	N372	0.45825	0.60952	0.55583
C179	0.39528	0.61376	0.09538	C374	0.43923	0.62247	0.57103
C180	0.41084	0.64615	0.09783	C375	0.45398	0.65483	0.57271
C181	0.36285	0.59672	0.0979	C376	0.4068	0.60432	0.58177
C182	0.39458	0.66145	0.10612	C377	0.43696	0.66913	0.58538
C184	0.34648	0.61199	0.10557	C379	0.38967	0.61862	0.59378
C186	0.36216	0.64451	0.11065	C381	0.40456	0.6512	0.59578
C189	0.34481	0.66075	0.11821	C384	0.38656	0.66646	0.60981
C190	0.35997	0.69173	0.13047	C385	0.40123	0.69685	0.63236
Zn195	0.52519	0.54309	0.10202	Zn390	0.57127	0.55493	0.5665

Table S5. Summary of experimental BET surface areas, and total pore volumes of as-synthesized M(salen)-COFs obtained by one-step synthesis and two-step synthesis.

**COFs**	**Experimental S_{BET} (m^2 g^{-1})**	**Total pore volumes (cm^3 g^{-1})**
Zn-TAPB-COF-1	565	0.990
Zn-TAPB-COF-2	338	0.989
ZnZn-TAPB-COF-1	666	0.990
ZnZn-TAPB-COF-2	649	0.990
Zn-TAPT-COF-1	556	0.989
Zn-TAPT-COF-2	320	0.989
ZnZn-TAPT-COF-1	656	0.990
ZnZn-TAPT-COF-2	576	0.989
Co-TAPB-COF-1	500	0.990
Co-TAPB-COF-2	555	0.990
Co-TAPT-COF-1	753	0.990
Co-TAPT-COF-2	754	0.989

Table S6. EXAFS fitting parameters at the Zn *K*-edge for Zn-TAPB-COF-1, ZnZn-TAPB-COF-1 and Co *K*-edge for Co-TAPT-COF-1, respectively.

Sample	Shell	***CN***[a]	***R*****(Å)**[b]	**σ^2(Å^2)**[c]	**ΔE_0(eV)**[d]	***R* factor**
Zn foil	Zn-Zn	6*	2.64±0.01	0.014±0.001	2.38±0.22	0.006
ZnO	Zn-O	3.5±0.5	1.95±0.01	0.003±0.0041	5.28±2.16	0.009
	Zn-Zn	12.2±3.7	3.25±0.01	0.01±0.0010	-2.46±0.9	
Zn-TAPB-COF-1	Zn-O	2.6±0.88	1.95±0.01	0.009±0.005	4.57±4.37	0.014
	Zn-N	2.09±1.21	2.14±0.01	0.01±0.01	7.06±10.67	
ZnZn-TAPB-COF-1	Zn-O	2.0±1.5	1.94±0.01	0.007±0.003	5.03±5.2	0.006
	Zn-N	2.3±1.3	2.10±0.01	0.007±0.003	-1.74±8.8	
Co foil	Co-Co	12*	2.48±0.01	0.005±0.0004	6.6±0.5	0.003
CoO	Co-O	5.6±1.7	2.11±0.01	0.01±0.006	0.5±3.4	0.009
	Co-Co	12.5±1.9	2.99±0.01	0.01±0.001	-3.9±1.4	
Co_3O_4	Co-O	4.3±0.5	1.91±0.01	0.002±0.001	-5.9±1.7	0.006
	Co-Co	4.2±1.7	2.86±0.01	0.004±0.002	-7.6±3.4	
	Co-Co	8.1±3.6	3.35±0.01	0.006±0.003	-9.5±2.6	
Co-TAPT-COF-1	Co-O/N	5.9±1.1	1.86±0.01	0.004±0.002	-9.2±2.9	0.02

[a]*CN*, coordination number; [b]*R*, distance between absorber and backscatter atoms; [c]σ^2, Debye-Waller factor to account for both thermal and structural disorders; [d]ΔE_0, inner potential correction; *R* factor indicates the goodness of the fit. $S0^2$ was fixed to 0.950 for Zn and 0.78 for Co, according to the experimental EXAFS fit of Zn foil and Co foil by fixing CN as the known crystallographic value. Fitting range: 3.0 ≤ k (/Å) ≤ ~12.4 and 1.5 ≤ R (Å) ≤ 3.0.

Table S7. Photocatalytic CO_2RR performance in different conditions with Co-TAPT-COF-1 as photocatalyst.

Catalysts	Solvents (mL)	Photosensitizer (mg)	H_2 Rate (mmol g^{-1} h^{-1})	CO Rate (mmol g^{-1} h^{-1})
Co-TAPT-COF-1	CH_3CN/TEOA/H_2O 70/20/10	10	0.34	1.19
		20	1.91	3.39
		30	3.42	4.61
		40	6.43	8.10
		60	11.31	8.39
	CH_3CN/TEOA/H_2O 75/20/5	60	10.00	4.86
	CH_3CN/TEOA/H_2O 60/20/10		8.76	6.87

Table S8. H_2/CO ratios obtained from CO_2 photoreduction with as-synthesized M(salen)-COFs.

Entry	Sample	n(H_2)/n(CO)	Entry	Sample	n(H_2)/n(CO)
1	ZnZn-TAPB-COF-1	30:1	7	ZnZn-TAPB-COF-2	17:1
2	Zn-TAPT-COF-1	29:1	8	Zn-TAPT-COF-2	16:1
3	ZnZn-TAPT-COF-1	25:1	9	Co-TAPT-COF-2	24:19
4	Zn-TAPB-COF-2	25:1	10	Co-TAPT-COF-1	11:8
5	ZnZn-TAPT-COF-2	23:1	11	Co-TAPB-COF-1	1:1
6	Zn-TAPB-COF-1	20:1	12	Co-TAPB-COF-2	4:3

Table S9. Performance comparison of Co-TAPT-COF-1 with reported photocatalysts.

**Catalysts**	**active sites**	**solvent**	**CO rate (mmol g^{-1} h^{-1})**	**Ref.**
Re-COF	Re	CH_3CN/ TEOA	0.75	*J. Am. Chem. Soc*. **2018**, *140*, 14614.
DQTP-Co-COF	Co	CH_3CN/ TEOA	1.02	*Appl. Catal. B: Environ*. **2019**, *254*, 624.
COF-367 NSs	Co-N_4	0.1 M $KHCO_3$/AA	10.162	*J. Am. Chem. Soc*. **2019**, *141*, 17431
Ni-TpBpy-COF	Ni	MeCN/H_2O/TEOA	0.966	*J. Am. Chem. Soc*. **2019**, *141*, 7615
N-CP-D	Co-bipy	CH_3CN/ H_2O/ TEOA	2.247	*Nat. Commun*. **2020**, *11*, 1149
Re-Bpy-COF	Re	CH_3CN/ TEOA	1.04	*Chem. Sci*. **2020**, *11*, 543
Co-Py-CON	$[Co(Bpy)_3]^{2+}$	CH_3CN/ H_2O/ TEOA	1.683	*Chem. Mater*. **2020**, *32*, 9107
NiPc–Co-POP	Ni–N_4	CH_3CN/ H_2O/ TEOA	1.942	*Adv. Mater*. **2021**, *33*, 2101568
Re-HOF-25	Re	CH_3CN/ TIPA	3.03	*Angew. Chem. Int. Ed*. **2021**, *60*, 8983
NUST-6	Por-H	CH_3CN/ H_2O/ TEOA	0.00762	*J. Am. Chem. Soc*. **2022**, *144*, 5728
Co-SA-COF	Co-SA	MeCN / TEOA	1.48	*ACS Sustain. Chem. Eng*. **2021**, *9*, 13376
Co-TAPT-COF-1	CoN_2O_2	CH_3CN/ H_2O/ TEOA	8.39	**This work**

Table S10. The calculated free energies (eV) on Co-TAPT-COF, Zn-TAPT-COF and ZnZn-TAPT-COF.

**Pathway**	**Co**	**Zn**	**ZnZn**
*+CO_2	0.00	0.00	0.00
*CO_2H	0.75	2.25	2.02
*CO	0.13	0.43	0.44
*+CO	0.15	0.15	0.15

Table S11. The CBM values, VBM values and bandgaps of the Co-TAPT-COF-1, Co-TAPT-COF-2 and Co-TAPB-COF-1 calculated from UV-visible spectra, Mott-Schottky plots and UPS.

**Sample**	**Eg (eV)**	**CBM *vs.* NHE (pH = 7)**	**VBM *vs*. NHE (pH = 7)**
Co-TAPT-COF-1	2.38	-1.29	1.09 (1.07 in UPS)
Co-TAPT-COF-2	2.33	-1.34	0.99
Co-TAPB-COF-1	2.62	-0.95	1.67 (1.68 in UPS）
