# Supplementary material for: Photocatalytic CO2 reduction to syngas using metallosalen covalent organic frameworks
Source: Nat Commun. 2023 Nov 1;14:6971. doi: 10.1038/s41467-023-42757-7 (PMC10620383; doi:10.1038/s41467-023-42757-7)
Supplement: Supplementary file 3 — Description of Additional Supplementary Files [file 41467_2023_42757_MOESM3_ESM.pdf]

Description of Additional Supplementary Files

File Name: Supplementary Data 1
Description: CIF files of M(salen)-COFs constructed using the Materials Studio software.
